# Identification of hepatic fibrosis inhibitors through morphometry analysis of a hepatic multicellular spheroids model

**DOI:** 10.1038/s41598-021-90263-x

**Published:** 2021-05-25

**Authors:** Yeonhwa Song, Sanghwa Kim, Jinyeong Heo, David Shum, Su-Yeon Lee, Minji Lee, A-Ram Kim, Haeng Ran Seo

**Affiliations:** 1 Cancer Biology Laboratory, Institut Pasteur Korea, 16, Daewangpangyo-ro 712 beon-gil, Bundang-gu, Seongnam-si, 13488 Gyeonggi-do Republic of Korea; 2 Screening Discovery Platform, Institut Pasteur Korea, 16, Daewangpangyo-ro 712 beon-gil, Bundang-gu, Seongnam-si, 13488 Gyeonggi-do Republic of Korea; 3 Division of Bio-Medical Science and Technology, University of Science and Technology, Deajeon, 34113 Republic of Korea

**Keywords:** Cancer, Drug discovery, Gastroenterology

## Abstract

A chronic, local inflammatory milieu can cause tissue fibrosis that results in epithelial-to-mesenchymal transition (EMT), endothelial-to-mesenchymal transition (EndMT), increased abundance of fibroblasts, and further acceleration of fibrosis. In this study, we aimed to identify potential mechanisms and inhibitors of fibrosis using 3D model-based phenotypic screening. We established liver fibrosis models using multicellular tumor spheroids (MCTSs) composed of hepatocellular carcinoma (HCC) and stromal cells such as fibroblasts (WI38), hepatic stellate cells (LX2), and endothelial cells (HUVEC) seeded at constant ratios. Through high-throughput screening of FDA-approved drugs, we identified retinoic acid and forskolin as candidates to attenuate the compactness of MCTSs as well as inhibit the expression of ECM-related proteins. Additionally, retinoic acid and forskolin induced reprogramming of fibroblast and cancer stem cells in the HCC microenvironment. Of interest, retinoic acid and forskolin had anti-fibrosis effects by decreasing expression of α-SMA and F-actin in LX2 cells and HUVEC cells. Moreover, when sorafenib was added along with retinoic acid and forskolin, apoptosis was increased, suggesting that anti-fibrosis drugs may improve tissue penetration to support the efficacy of anti-cancer drugs. Collectively, these findings support the potential utility of morphometric analyses of hepatic multicellular spheroid models in the development of new drugs with novel mechanisms for the treatment of hepatic fibrosis and HCCs.

## Introduction

Hepatic fibrosis results as a consequence of a pattern of severe inflammation that leads to the excessive accumulation of extracellular matrix (ECM) proteins. Advanced liver fibrosis results in cirrhosis and is directly related to the high mortality of cirrhosis^1^. Because liver transplantation is currently the only treatment option for patients with advanced liver fibrosis and cirrhosis, there is an urgent need for the development of effective anti-fibrotic agents for the treatment of hepatic fibrosis.

Indeed, despite the high prevalence of liver fibrosis, there are no approved therapies, potentially because liver fibrosis represents a diverse state with numerous potential causes and complications. The main causes of liver fibrosis are chronic hepatitis virus infection^2^, alcohol abuse^3^, drug-induced liver injury (DILI)^4^, cholestasis^5^, and non-alcoholic steatohepatitis (NASH)^6^. These causal pathways are related in that they each contribute to a sustained pattern of hepatic injury and inflammation, which eventually contributes to the development of fibrotic tissue.

There are four basic cell types that reside in the liver. The specialized parenchymal cells are the hepatocytes, and the non-parenchymal cell types are principally liver sinusoidal endothelial cells (ECs), kupffer cells, and hepatic stellate cells (HCSs)^7^. Liver tissues with severe fibrosis suffer from sustained hepatocyte damage and the resulting production of fibrogenic cytokines^8^ (such as TGF-β1, angiotensin II), which induce the activation of non-parenchymal cells such as hepatic stellate cells^9^ and ECs^10^. Activation of HSCs^11,12^ and endothelial-to-mesenchymal transition (EndMT) of ECs^10,13^ leads to deposition of uncommonly large amounts of ECM that contributes to liver fibrosis. To slow or reverse fibrosis, prior work in the development of anti-fibrotic drugs has aimed to inhibit the production of fibrogenic cytokines by reprograming of activated HCSs and/or ECs and to prevent the deposition of ECM proteins. However, there remains a strong need for the development of sophisticated in vitro methodologies that are reflective of the complex microenvironment observed in liver fibrosis to identify candidate anti-fibrotic drugs for advancement.

In our previous work, we reported the reciprocal crosstalk between cancer cells and stromal cells (fibroblast, vascular endothelial cells, and hepatic stellate cells) in a spheroid model system, which increases the expression of ECM molecules and the expression of proteins related to both epithelial-mesenchymal transition (EMT) and EndMT in multicellular tumor spheroids (MCTSs) relative to monolayer culture systems. Additionally, we found that the reciprocal crosstalk between parenchymal cells and non-parenchymal cells in the spheroid system more efficiently induces transformation of HSCs and EC cells than a similar monolayer culture system^9,14,15^. 3D multicellular spheroids are inherently better able to capture the elements of heterogeneity, hypoxia, nutrient penetration, pH, and metabolite gradients observed in vivo, and have thus been increasingly used as a model of cell proliferation and transformed animal models^16,17^. We hypothesized that the MCTSs model would be an appropriate system to mimic the behavior of the EMT, EndMT and the liver fibrosis in vivo*.* We have developed an automated imaging platform that systematically analyzes dynamics in cell culture based on a state-of-the-art fluorescence imaging platform and high-end image analysis technology^18,19^.

In this study, we applied MCTSs for high-throughput screening (HTS) to identify compounds that may be able to treat liver fibrosis. To reflect extensive fibrosis in vitro, human HCC cells were grown together with human fibroblasts (WI38), human HSCs (LX2), and human umbilical endothelial cells (HUVEC) in MCTSs. Subsequently, we performed HTS with FDA-approved drugs to identify compounds that specifically reversed the fibrotic properties in MCTSs.

## Materials and methods

### Cell culture

Human liver cancer cells; Huh7 and SNU449 were obtained from the Korean Cell Line Bank (Seoul, Korea) and maintained in Roswell Park Memorial Institute medium (RPMI 1640; Welgene, Daegu, Korea). The human immortalized hepatocyte Fa2N-4 cell line were purchased from Xenotech (Lenexa, KS, USA), and maintained in serum-containing plating mediun (Xenotech) at first. After cell attachment in the plate, the medium was replaced with supporting culture medium (Xenotech). Human hepatic stellate cells (HSCs), LX2, was purchased from Merck Millipore (Darmstadt, Germany), and maintained Dulbecco`s modified Eagle`s medium (DMEM; Welgene) containing 2% fetal bovine serum (FBS; Gibco, Grand Island, NY, USA) and 1% penicillin–streptomycin (P/S; Gibco). One of HCC cell lines, HepG2 and WI38 human fibroblast cell line were obtained from ATCC (Manassas, VA, USA). These cell lines were maintained in minimum essential media (MEM; Welgene) supplemented with 10% FBS and 1% P/S. the Human umbilical vein endothelial cells (HUVECs) was purchased from PromoCells (Heidelberg, Germarny), and cultured in endothelial basal medium with supplementary reagents from PromoCells (Heidelberg, Germarny). All cells were maintained at 37 °C in a humidified incubator with 5% CO_2_.

### Compounds

Forskolin (SC-3562, Santa Cruz Technology)**,** Retinoic acid (H7779, Sigma-Aldrich), Pirfenidone (P2116, Sigma-Aldrich), Nintedanib (S1010, Selleck Chemical), Pregnenolone succinate (700142P, Sigma-Aldrich), Rosiglitazone (R2408, Sigma-Aldrich), Ursocholanic acid (C7628, Sigma-Aldrich), Sorafenib (sc-220125, Santa Cruz Technology), AM580 (A8843, Sigma-Aldrich), TTNPB (S4627, Selleck Chemical), NKH477 (N3290, Sigma-Aldrich), Irsogladine maleate (sc-201190, Santa Cruz Technology), Pomiferin (CFN93047, Chemfaces), Tretinoin (PHR1187-3X, Sigam-Aldrich), Tazarotene (23559, Cayman Chemical, Ann Arbor, MI, USA).

### Generation of HCC tumor spheroids and multicellular tumor spheroids (MCTSs)

To generate HCC tumor spheroids, cells were seeded at a density of 6 × 10^3^ cells/well in 96-well round bottom ultra-low attachment microplates (Corning Life Sciences, Amsterdam, Netherlands). For MCTS generation, HCC cells were seeded at a density of 3.3 × 10^3^ cells/well, and LX2, WI38, and HUVEC cells were seeded at a density of 0.9 × 10^3^ cells/well/cell line together in 96-well round bottom ultra-low attachment microplates. For MCHS generation, Fa2N-4 cells were seeded at a density of 0.9 × 10^3^ cells/well/cell line together in 96-well round bottom ultra-low attachment microplates. The plates were incubated for 3 days at 37 °C in a humidified incubator with 5% CO_2._ After 3 days, anti-fibrosis drugs were added and incubated for an additional 2 days.

### Microarray analysis

Global gene expression analysis was performed using Affymetrix GeneChip Human Gene 2.0 ST Arrays. Total RNA from HCC spheroids and MCTS was isolated using the RNeasy Mini kit (Qiagen, Hilden, Germany). RNA quality was assessed using an Agilent 2100 Bioanalyser using the RNA 6000 Nano Chip (Agilent Technologies), and the quantity was determined using a Nanodrop-1000 Spectrophotometer (Thermo Fisher Scientific). We used 300 μg of each RNA sample as input for the Affymetrix procedure, as recommended in the manufacturer’s protocol (http://www.affymetrix.com). Briefly, 300 ng of total RNA from each sample was converted to double-stranded cDNA using a random hexamer incorporating a T7 promoter, and amplified RNA (cRNA) was generated from the double-stranded cDNA template though an in vitro transcription (IVT) reaction and purified using the Affymetrix sample cleanup module. cDNA was regenerated through randomly primed reverse transcription using a dNTP mix containing dUTP. The cDNA was then fragmented by uracil-DNA glycosylase (UDG) and apurinic/apyrimidinic endonuclease (APE1) restriction enzymes, and end-labeled via a terminal transferase reaction incorporating a biotinylated dideoxynucleotide. Fragmented end-labeled cDNA was hybridized to the GeneChip Human Gene 2.0 ST array for 17 h at 45 °C and 60 rpm, as described in the Gene Chip Whole Transcript (WT) Sense Target Labeling Assay Manual (Affymetrix). After hybridization, the chips were stained and washed in a Genechip Fluidics Station 450 (Affymetrix) and scanned using a Genechip Array scanner 3000 7G (Affymetrix). The expression intensity data were extracted from the scanned images using Affymetrix Command Console software, version 1.1, and stored as CEL files.

### Western blot analysis

Cell pellets were collected by centrifuged and lysed in a lysis buffer (Thermo Fisher Sciences, MA, and USA). The supernatants were collected by centrifuged at 12,000 rpm for 20 min. The proteins amounts were analyzed using the bicinchonic acid (BCA) methods following manufacture’s instruction (Thermo Fisher Sceince, Ma, USA). Equal amounts of protein were separated on 8 or 10% SDS-PAGE gels, after electrophoresis, the proteins were transferred onto a nitrocellulose (NC) membrane (Pall, Port Washington, NY, USA). Membranes were blocked with 5% skim milk (BD Bioscience) for 30 min at R.T. After blocking, they were immune-blotted with specific primary antibodies following to: N-cadherin (ab76057, 1:500), E-cadherin (ab40772, 1:200), vimentin (ab8978, 1:3000), human alpha smooth muscle actin (α-SMA, ab32575, 1:3000), human fibroblast activation protein (FAP, ab28244, 1:1000) and cleaved caspase-3 (ab2302, 1:1000) were purchased from Abcam (Cambridge, MA, USA). Snail (3879s, 1:1000), Smad2/3 (3102, 1:1000), p-Smad2 (Ser465/467) (3108, 1:1000), p-Smad3 (Ser423/425) (9520, 1:1000) and CD31 (3528, 1:1000) were purchased from Cell Signaling Technology (Danvers, MA, USA). Collagen I ((NB600-408, 1:1000) was obtained from Novus Biologicals (Centennial, CO, USA), and CD133/1 (AC133, 130-090-422, 1:100) was purchased from Miltenyi Biotec (Bergisch Gladbach, Germany). All primary antibodies were incubated for 16 h at 4 °C. After washing, the blots were incubated with corresponding anti-rabbit and anti-mouse IgGs conjugated with horseradish peroxidase (Cell Signaling Technology) for 1 h. Immuno-reactive proteins were detected using ECL reagent (Thermo Fisher Scientific). β-actin was purchased from sigma-Aldrich (St Louis, MO, USA) and was used as control of each samples.

### High-throughput screening

A library of 4,763 compounds was assembled from Tocris Bioscience (Avonmouth, Bristol, UK), Selleck Chemicals /8Houston, TX, USA), LOPAC (St Louis, MO, USA), and Prestwick Chemical (Washington, DC, USA) and screened in the MCTS model at a final concentration of 10 µM in 0.5% DMSO (v/v) (Sigma-Aldrich,). All cells were seeded at a density of 6 × 10^3^ cells/well into 96-well round bottom ultra-low attachment microplates. The plates were then incubated at 37 °C in a humidified atmosphere of 5% CO_2_ for 3 days. For the testing of compounds, a 2 μL sample of each compound was transferred into an intermediate 384-well polypropylene plate (Greiner Bio-one, Monroe, NC, USA) using a liquid handler (Apricot Personal Pipettor; Apricot Design, Covina, CA, USA). The compounds were mixed with 78 μL complete medium per well. Subsequently, a 20 μL sample of each compound was dispensed into each well of a 96-well assay plate. The plates were then incubated at 37 °C in a humidified atmosphere of 5% CO_2_ for 7 days. Sorafenib was added as a positive control into each assay plate at its IC_50_ concentration as a low control and 0.5% DMSO (v/v) was were as a high control. After 10 days, spheroid images were acquired using a high-content screening system. The size of spheroids was measured using a self-developed algorithm. Hit compounds were selected using a threshold based on 3σ (standard deviation) from the IC_50_ of sorafenib.

### Staining of MCTSs

After generating MCTSs for 3 days, spheroids were treated with 5 µM Retinoic acid (Sigma-Aldrich) or 5 µM Forskolin (Santa Cruz Biotechnology, Dallas, TX, USA) for additional 2 days, spheroids were collected and transferred to 384-well microplates (781091, Greiner Bio-one). Spheroids were fixed with 4% paraformaldehyde; PFA (Biosesang, Seoul, Korea). For staining F-actin, Alexa Fluor 488 phalloidin (A12379; Invitrogen, Eugene, OR, USA) was incubated for 1 h at R.T. After wash step with DPBS for 3 times, Hoechst33342 (H3570; Invitrogen) with DPBS was incubated for 10 min at R.T. After being washed 3 times, images were obtained with Operetta CLS system (Perkin-Elmer, Waltham, MA, USA).

### Anti-fibrosis compound validation 2D model

Hepatic stellate cells (LX2) or Endothelial cells (HUVECs) were seeded at 2.5 × 10^3^ cells/well in 384-well microplate and incubation for 16 h at 37 °C in a humidified incubator of 5% CO_2_. LX2 and HUVECs were treated with 20 ng/ml recombinant human TGF-β1 (100-21C, Peprotech, Cranbury, NJ, USA) for activation, and compounds were treated for 48 h simultaneously. After incubation, cells were fixed with 4% PFA for 10 min at R.T. and incubated with primary antibodies against α-SMA (ab32575, 1:3000; Abcam) for 16 h at 4 °C. After being washed with DPBS for 3 times, the samples were incubated with secondary antibodies with fluorescence including Alexa Fluor 488 (A11008, 1:500; Invitrogen) and Alexa Fluor Phalloidin 633 (A22284, 1:100; Invitrogen) for 1 h at R.T. After washing with DPBS, the images were detected using Operetta CLS system and analyzed by Harmony software (Perkin-Elmer).

### Doxorubicin penetration in multicellular tumor spheroids (MCTSs)

After generating to MCTSs, 5 μM Retinoic acid or 5 μM Forskolin were added into MCTs for 2 days. After 2 days, 10 μM doxorubisin (Sigma-Aldrich) were additionally treated into incubated MCTS. The image acquisition were obtained by Operetta CLS with a 530 to 560 nm excitation and a 573 to 647 nm emission filter set.

### Statistical analysis

All experiments were performed in duplicate. The results are expressed as the mean ± standard derivation (SD). Statistical analysis was performed using Student’s *t*-test.

### Consent for publication

All authors read and approved the final manuscript for publication.

## Results

### Establishment of multicellular tumor spheroids (MCTSs) that recapitulate important elements of hepatic fibrosis for high-throughput screening of potential liver fibrosis inhibitors

**Figure 1:**
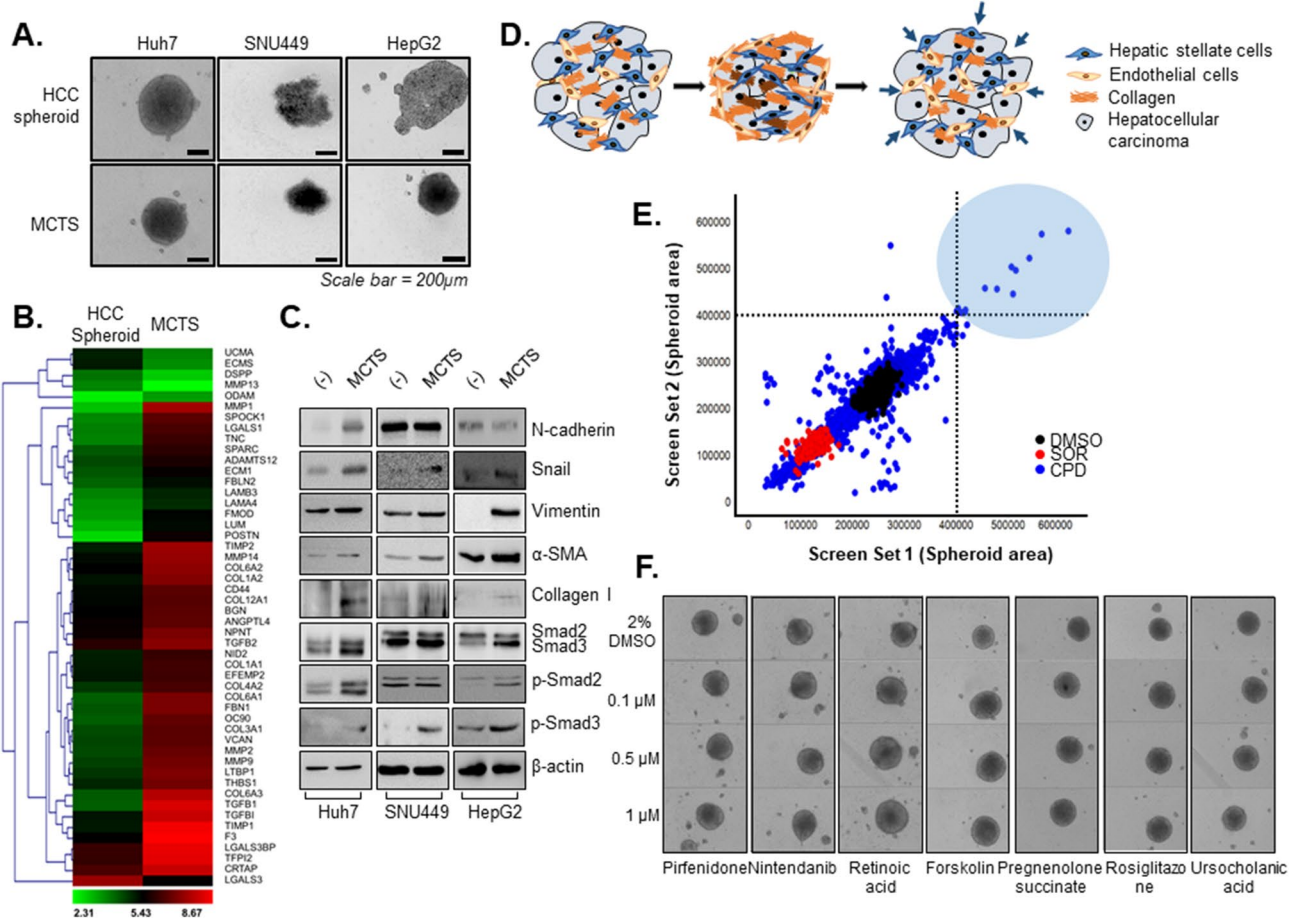
Establishment of multicellular tumor spheroids (MCTSs) as a model of liver fibrosis and screening for inhibitors of fibrosis. **(A)** HCC spheroids and MCTS were developed using an HCC cell line (Huh7, SNU449, or HepG2) and stromal cells (LX2, WI38, and HUVEC). **(B)** Microarray analysis of Huh7 spheroid and Huh7 MCTS. **(C)** Mesenchymal, EMT, ECM, TGF-β1 signaling-related protein expression in HCC spheroids and HCC-MCTSs (Huh7, SNU449, or HepG2). **(D)** Scheme of 3D phenomic-based assay model using Huh7.5 cell in MCTS in vitro. **(E)** The scatterplot analysis of positive (red; sorafenib IC_50_), negative (black: 0.5% DMSO) control, and 4763 compounds comprised of FDA-approved drugs from a compound library. **(F)** MCTS treated with 0.1, 0.5, or 1 µM HIT compounds identified in the screen. All images were obtained using the Operetta CLS system.

In our previous study, we found that the interaction between HCC cells and various non-parenchymal cells affected the compactness of the spheroids as well as cell migration through accumulation of collagen and EMT-related proteins^11^. In order to generate a fibrosis model in vitro, various HCC cell lines (Huh7 cells, SNU449 cells, and HepG2 cells) were grown together with fibroblasts (WI38), hepatic stellate cells (LX2), and endothelial cells (HUVEC) in MCTS models. Despite the fact that both SNU449 cells and HepG2 cells innately formed loose aggregates, these cells acquired the rigidness of the spheroids following co-culture with stromal cells in spheroids. Similarly, although Huh7 cells formed a relatively solid spheroids, co-culture with stromal cells enhanced rigidness in spheroids (Fig. 1A). This result showed that crosstalk between stromal cells and HCC cells in MCTS models was an important determinant of rigidness of spheroids, emphasizing the importance of culturing these cells as a system rather than as individual components.

Next, gene expression profiling was performed on the MCTS model systems to compare against the expression profiles observed in tumor spheroids. In the MCTS models, genes that were involved in the production of ECM structural constituents were significantly enriched. In particular, we found increased relative expression of *MMP1*, *COL6A1*, *COL6A3*, and *TGFB1* in MCTS relative to tumor spheroids [Fig. 1B].

Because the process of EMT leads to organ fibrosis, we compared the expression of mesenchymal markers such as vimentin, α-smooth muscle actin (α-SMA), Snail, and N-cadherin between the MCTS models and tumor spheroids. Mesenchymal markers were generally upregulated in all MCTS models relative to tumor spheroids alone. In particular, Snail, Vimentin, α-SMA, Collagen I and p-Smad2 were significantly elevated in all MCTS model. Because TGF-β1, which is a critical regulator of fibrosis, stimulates the EMT and EndMT processes through the activation of Smad, we measured the expression and activation of Smad2 and Smad3. Expression of Smad2/3 and relative abundance of p-Smad2, p-Smad3 were upregulated in Huh7-, HepG2-MCTSs. SNU449 MCTSs also displayed upregulated p-Smad3 expression (Fig. 1C and Supplementary Fig. 1).

The MCTSs provided a useful in vitro model of liver fibrosis, where increased size of spheroids, which results from the loss of tight cross-linking among cells, indicates decreased expression of fibrosis-related proteins and thus provides a reliable morphometric indication of the reversal of liver fibrosis (Fig. 1D). Next, we sought to establish a MCTS-based drug screening platform for the evaluation of potential liver fibrosis inhibitors. To obtain reproducible results, we plated single, homogenously sized and configured spheroids in 384-well plates for HTS screening.

A library comprised of 4,763 drug compounds with known molecular targets was tested for potentially promising candidates as inhibitors of fibrosis. All compounds were screened at an initial concentration of 10 µM in duplicate to confirm the reproducibility of the observed effects. A correlation coefficient of 0.89 for replicate screens indicated that the assay was reliable (Fig. 1E). In that screening, we identified 12 positive compounds (HITs) including four compounds involved in the cAMP/PKA pathway, five retinoic acid analogs, an anti-diabetic drug, a regulator of cholesterol, and a NMDA receptor modulator (Table 1).

**Table 1:** List of HITs as anti-fibrotic compounds.

HIT compounds	Function
Irsogladine maleate	PDE4 inhibitor; antiulcer agent
Forskolin	Adenylyl cyclase activator
NKH 477	Adenylyl cyclase activator
Pomiferin	PDE5 inhibitor
Retinoic acid	Endogenous retinoic acid receptor agonist
TTNPB	Retinoic acid analog; RAR agonist
AM 580	Retinoic acid agonist
Tretinoin	All-*trans* retinoic acid
Tazarotene	Retinoic acid receptor alpha (RAR-α)
Rosiglitazone	Anti-diabetic medication
Ursocholanic acid	Cholesterol absorption
Pregnenolone succinate	NMDA receptor modulator

Because nintedanib and pirfenidone were recently authorized for the treatment of idiopathic pulmonary fibrosis, we evaluated the effects of both drugs on the size of HCC-MCTSs. Surprisingly, nintedanib and pirfenidone did not alter the morphology of HCC-MCTSs relative to control solvent (2% DMSO) (Supplementary Fig. 2). On the other hand, treatment with 10 µM concentration of the 12 HITs significantly increased of size of HCC-MCTSs, according to morphometric analyses, suggesting inhibition of fibrosis.

**Figure 2:**
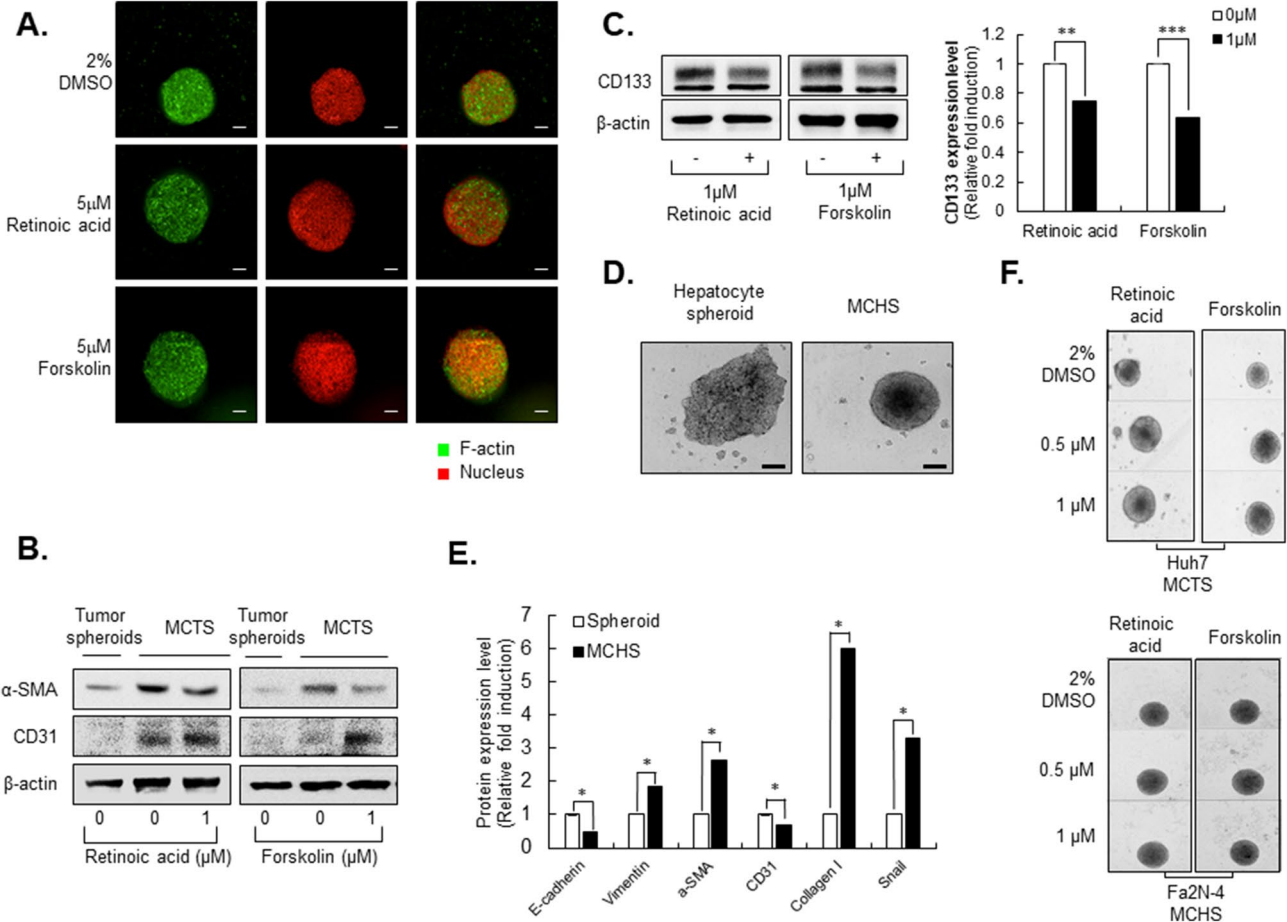
Reprogramming of liver fibrosis in the multicellular tumor spheroid (MCTS) model but not the multicellular hepatocyte spheroid (MCHS) model. **(A)** Structure through F-actin staining of MCTS treated with 5 µM of retinoic acid or forskolin. **(B)** Expression of mesenchymal-related marker (α-SMA), and an EndMT-related marker (CD31) in tumor spheroids or MCTSs with or without treatment with 1 µM retinoic acid or forskolin. **(C)** Expression of cancer stem cell-related marker (CD133) in MCTS with or without 1 µM retinoic acid or forskolin (left panel). The expression of CD133 was quantified (right panel). **(D)** Spheroid formation of Fa2N-4 spheroids or Fa2N-4 MCHSs. **(E)** Expression of EMT-related proteins (E-cadherin, Snail), mesenchymal-related proteins (vimentin, α-SMA), EndMT-related protein (CD31), and ECM-related protein (collagen I) in hepatocyte spheroids and MCHSs. **(F)** Spheroids treated with 0.5 or 1 µM retinoic acid and forskolin in MCTS (upper panel) and MCHS (bottom panel). All images were obtained using the Operetta CLS system. Data are expressed as means ± SD (n = 3). ^**^P < 0.01, and ^***^P < 0.001 compared to the control group.

Next, dose–response studies were performed to find the most effective compounds among the 12 HITs identified from HTS in Huh7.5 cell in MCTSs. We found that the retinoic acid analogs and modulators of cAMP/PKA pathway, particularly retinoic acid and forskolin, led to the most significant increases of HCC-MCTSs at concentrations as low as 0.1 µM, in a dose-dependent manner (Fig. 1F and Supplementary Fig. 3).

**Figure 3:**
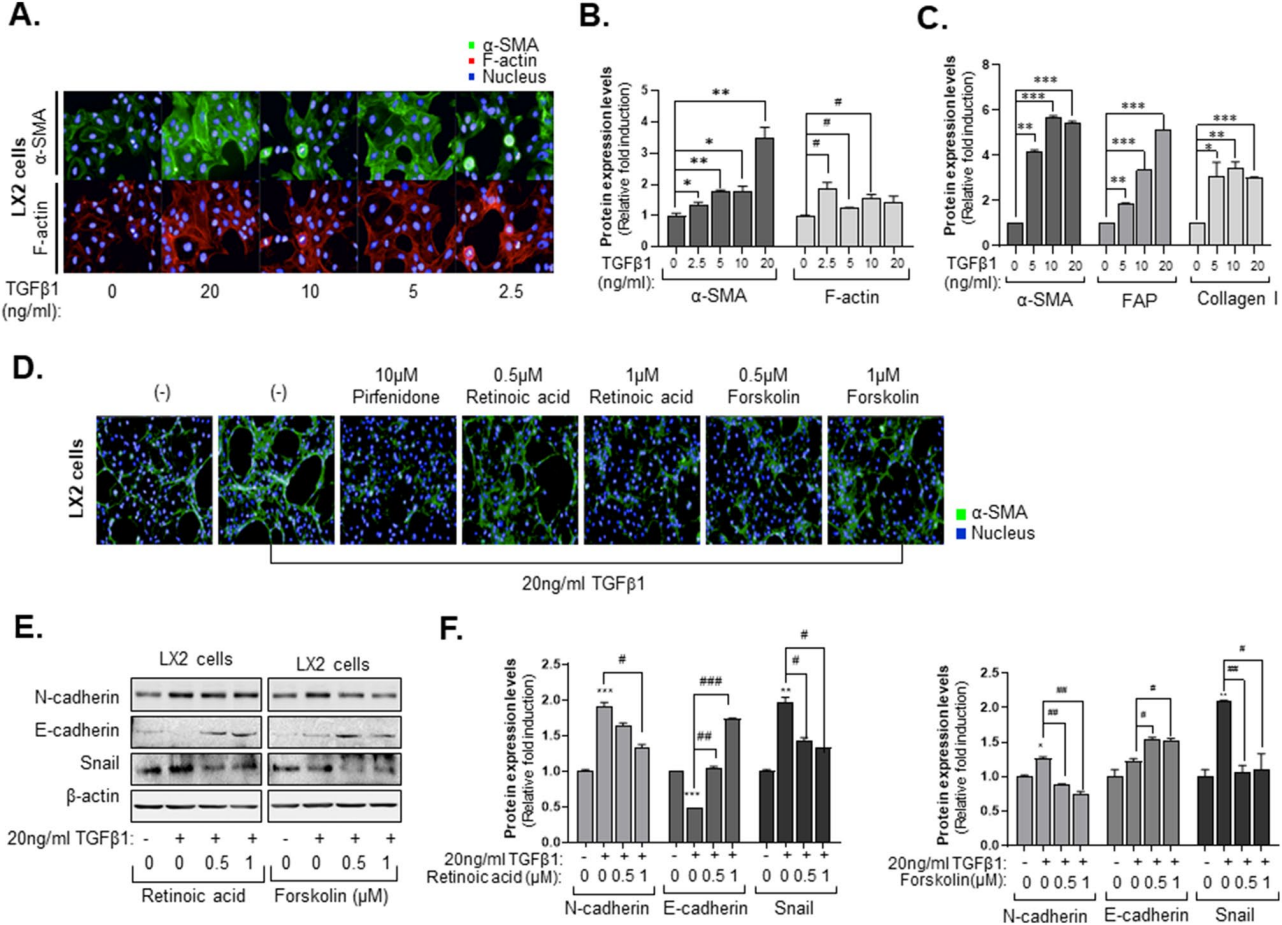
Anti-fibrosis effects of hit compounds in phenomic-based 2D culture of hepatic stellate cells. **(A)** Intensity of α-SMA and F-actin in hepatic stellate cells in a 2D culture system treated with TGF-β1. Cells were treated with 2.5, 5, 10, or 20 ng/ml TGF-β1 in 2% FBS media. **(B)** Calculated intensity of α-SMA and F-actin when LX2 cells were treated with TGF-β1 concentration. **(C)** The quantitative graph of mesenchymal-related proteins (α-SMA and FAP) and ECM-related protein (collagen 1) expressions depending on treating with TGF-β1 concentration in LX2 cells. **(D)** Representative images of α-SMA treated 10 µM pirfenidone, 0.5 µM or 1 µM retinoic acid and forskolin with 20 ng/ml TGF-β1 for 48 h in LX2 cells. **(E)** Expression of EMT-related protein (N-cadherin, E-cadherin, Snail) when LX2 cells were treated with 0.5 or 1 µM of retinoic acid or forskolin with or without 20 ng/ml TGF-β1 for 48 h by western blot assay. **(F) **The quantitative of western blot images was analyzed. All images were obtained using the Operetta CLS system. Data are expressed as means ± SD (n = 3). ^*^P < 0.05, ^**^P < 0.01, and ^***^P < 0.001 compared to the control group, ^*#*^*P* < 0.05, ^*##*^*P* < 0.01, and ^#*##*^*P* < 0.001 compared to the TGF-β1 treatment group.

### Retinoic acid and forskolin reversed EMT and EndMT in stromal cells in multicellular tumor spheroids (MCTSs), but not multicellular hepatocyte spheroids (MCHSs)

Generally, hepatic fibrosis is associated with upregulated expression of α-SMA via EMT and EndMT. To investigate the architectural changes observed in MCTSs following treatment with retinoic acid and forskolin, we used immunofluorescence assay to evaluate the expression of F-actin in spheroid structures. Interestingly, when 5 µM of retinoic acid and forskolin were added to MCTSs for 48 h, spheroid size was bigger than DMSO-treated MCTSs, without increasing of cell size, demonstrating loss of tight cell–cell interactions and decreasing F-actin intensity among the cells (Fig. 2A).

Western blot analysis also showed that elevation of α-SMA expression in MCTSs was sufficiently attenuated by treatment with 1 µM retinoic acid and forskolin, whereas expression of CD31 was increased under the same conditions. These results indicated that retinoic acid and forskolin inhibit the EndMT process as well as fibrotic properties in MCTSs (Fig. 2B, Supplementary Fig. 4). The retinoic acid analogs AM580 and TTNPB and the water-soluble forskolin derivative NKH477 also inhibited α-SMA expression in MCTSs, but did not alter expression of CD31, in contrast to retinoic acid and forskolin in MCTSs (Supplementary Fig. 5A).

**Figure 4:**
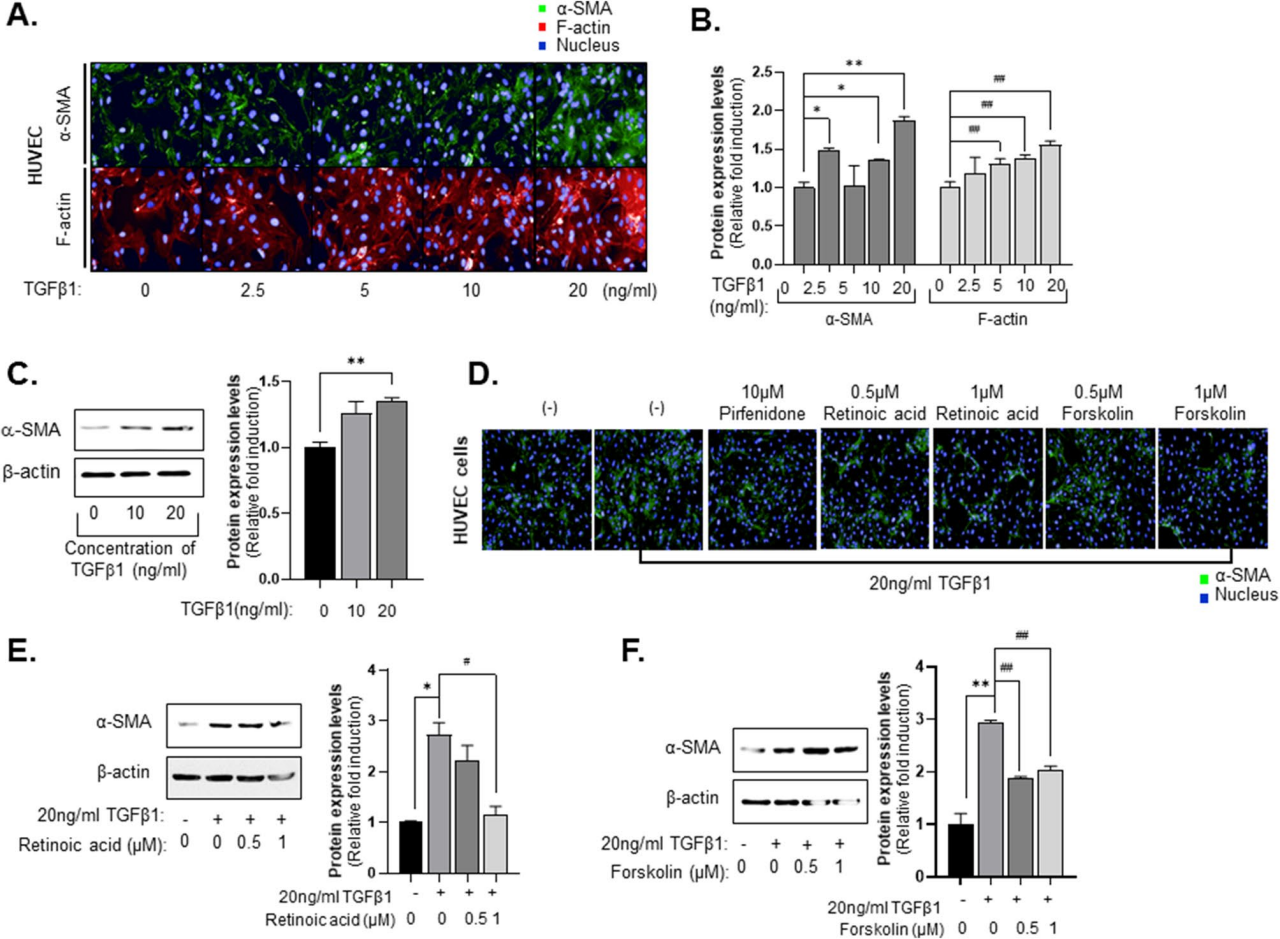
Anti-fibrosis effects of hit compounds in phenomic-based 2D assay of endothelial cells (HUVECs). **(A)** Intensity of α-SMA and F-actin in HUVEC cells in 2D culture system treating with TGF-β1. Cells were treated with 2.5, 5, 10, or 20 ng/ml TGF-β1 **(B)** Calculated intensity of α-SMA and F-actin when HUVEC cells were treated with TGF-β1 concentration. **(C)** α-SMA expression (left panel) and the quantitative of western blot image was analyzed (right panel) depending on treating with TGF-β1 concentration in HUVEC cells. **(D)** Representative images of α-SMA treated with 10 µM of pirfenidone, 0.5 µM or 1 µM of retinoic acid and forskolin with 20 ng/ml TGF-β1 for 48 h in HUVEC cells. **(E,F)** Expression of α-SMA when HUVEC cells were treated with 0.5 or 1 µM **(E)** Retinoic acid or **(F)** Forskolin. All images were obtained using the Operetta CLS system. Data are expressed as means ± SD (n = 3). ^*^P < 0.05 and ^**^P < 0.01 compared to the control group, ^*#*^*P* < 0.05 and ^*##*^*P* < 0.01 compared to the TGF-β1 treatment group.

**Figure 5:**
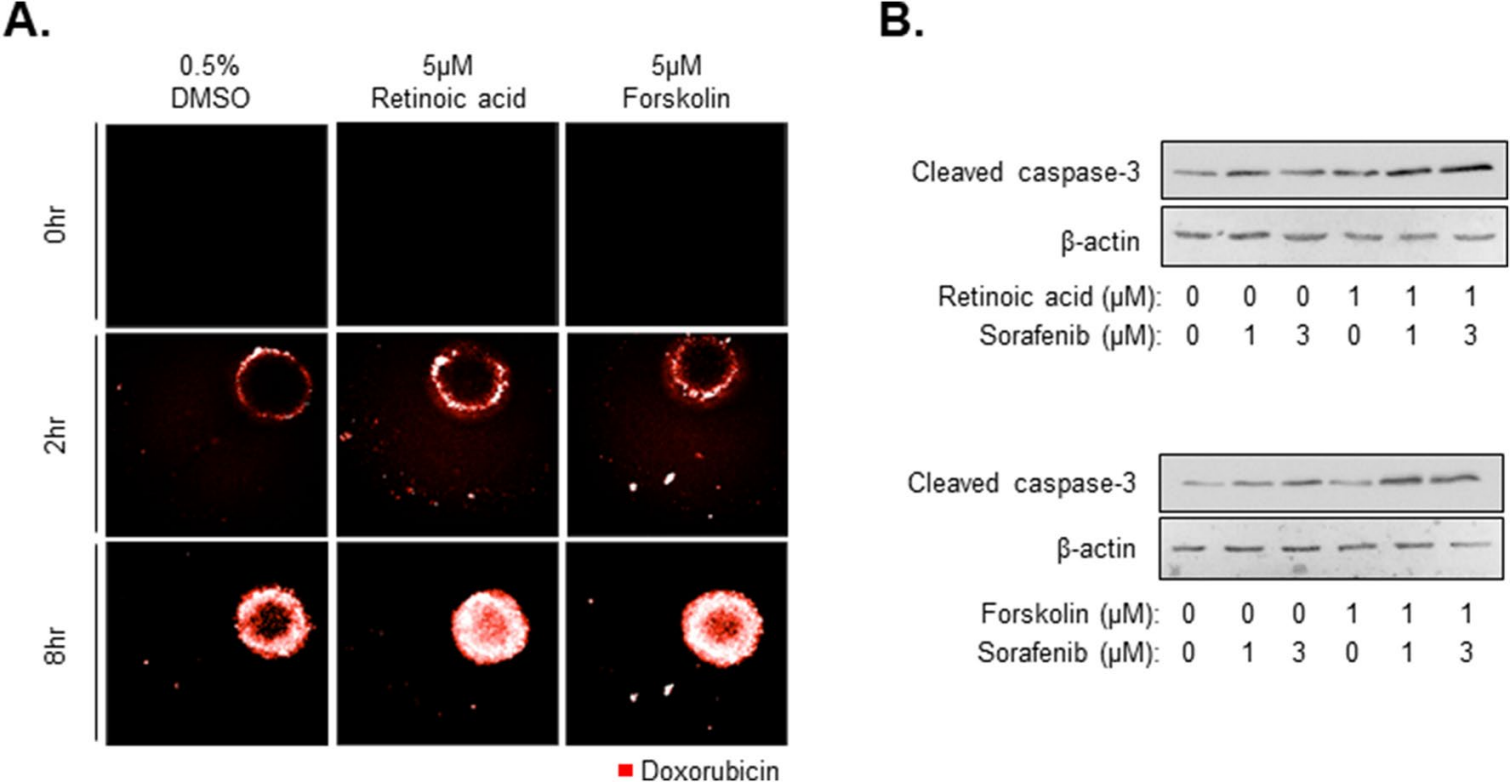
Improvement of anti-cancer activity through combination with anti-fibrosis compounds. **(A)** Drug penetration of MCTS treated with 5 µM Retinoic acid or Forskolin. After 48 h treatment with anti-fibrotic drugs, 10 µM doxorubicin was added to cells for 0, 2, or 8 h. **(B)** Expression of apoptosis-related protein, cleaved caspase-3, in MCTS with or without 1 or 3 µM sorafenib and 1 µM of retinoic acid or forskolin. All images were obtained using the Operetta CLS system.

Upregulation of CD133 facilitates EMT in various cancers^20–22^. Interestingly, expression of CD133 in MCTSs was also inhibited by treatment with 1 µM retinoic acid and forskolin (Fig. 2C). However, AM580, TTNPB and NKH477 did not inhibit CD133 expression as effectively as retinoic acid and forskolin (Supplementary Fig. 5B).

Next, we were curious whether the replacement of HCC cells with normal hepatocytes in the MCHSs would result in the same phenotypic effects. Instead of Huh7, we used Fa2N-4, a well-known normal hepatocyte cell line, to generate MCHSs. As shown in Fig. 2D, when stromal cells were mixed together, they formed compact spheroids. Similar to MCTSs, MCHSs also showed increasing expression of vimentin, α-SMA, collagen I, and Snail as well as decreasing E-cadherin and CD31 as was observed in the MCHS models (Fig. 2E and Supplementary Fig. 6). However, the hit compounds identified from HTS, retinoic acid and forskolin, did not change the size of spheroids created with normal hepatocytes. This suggests that MCTSs composed of HCCs exist in a severe inflammatory environment that is treatable with these compounds, making it a more suitable model for screening compounds than MCHSs with normal hepatocytes (Fig. 2F).

Liver fibrosis is a complex phenomenon orchestrated by numerous cellular actors in tumor microenvironments. These results suggested that retinoic acid and forskolin may inhibit hepatic fibrosis through reversing EMT and EndMT processes of stromal cells in MCTSs and suggested that an MCTS model-based morphometric screening approach may be a good strategy for the screening of novel effective therapies for fibrosis.

### Retinoic acid and forskolin depolarized hepatic stellate cells in a fibrotic environment

To confirm the potential efficacy of retinoic acid and forskolin in reprogramming activated HSCs, which are the main collagen-producing cells in liver fibrogenesis, we conducted cellular phenotype-based assays. Increasing production of α-SMA^23,24^ and F-actin stress fibers are associated with HSC activation when HSCs are stimulated with TGF-β1. To define distinctive morphometric signatures before and after TGF-β1 treatment, we focused on the expression pattern of F-actin and α-SMA after treatment with TGF-β1. Treatment with TGF-β1 increased the intense cytoplasmic α-SMA and F-actin of LX2 cells in a dose-dependent manner (Fig. 3A). When the intensity of α-SMA and F-actin were analyzed by Harmony 3.5.1 high-content imaging and analysis software, we found that treatment with 5 ng/ml TGF-β1 increased the intensity of α-SMA more than 1.5-fold compared to the control, whereas intensity of F-actin increased only slightly (Fig. 3B). Therefore, we selected α-SMA as a marker of fiberized hepatic stellate cells. Western blot analysis also displayed similar results in agreement with the cellular phenotype-based assays. Expression of fibroblast markers, α-SMA, fibroblast activation protein (FAP) and collagen I were increased after TGF-β1 treatment in LX2 cells (Fig. 3C and Supplementary Fig. 7).

Next, we measured the effects of retinoic acid and forskolin on TGF-β1-induced HSC activation using cellular phenotype-based assays. As expected, 1 µM retinoic acid and forskolin inhibited the expression of α-SMA after treatment with TGF-β1 in LX2 cells, with efficacy comparable to 10 µM pirfenidone, which served as our positive control. Particularly, Retinoic acid more efficiently induced reprogramming of activated HSCs activated than forskolin (Fig. 3D). Pirfenidone^25,26^ and nintedanib^27^, which are FDA-approved anti-fibrotic drug, inhibit TGF-β1 -induced fibrogenesis. However, in our system, the intensity of α-SMA was not decreased as much as 2% FBS-treated control when pirfenidone and nintedanib were treated at various concentrations (Supplementary Fig. 8). When 1 µM retinoic acid (Fig. 3E) and forskolin (Fig. 3F) were added with TGF-β1 to LX2 cells, EMT-related markers N-cadherin and Snail were inhibited, but E-cadherin was elevated, in contrast to EMT-related markers (Fig. 3E,F). Collectively, this phenotypic-based 2D assay system using LX2 cells appears to be an effective tool for validating anti-fibrosis compounds and suggested that retinoic acid and forskolin can reprogram activated hepatic stellate cells.

### Retinoic acid and forskolin suppress the EndMT process in HUVEC

In our previous studies, we established a visual phenomic screening platform to measure radiation-induced EndMT using HUVECs^28^. Herein, this technology was applied to measure TGFβ1-induced EndMT in HUVECs. HUVECs treated with TGF-β1 expressed increasing amounts of F-actin and cytoplasmic α-SMA in a dose-dependent manner (Fig. 4A). When the intensity of α-SMA and F-actin were analyzed by Harmony 3.5.1 high-content imaging and analysis software, we found that treatment with 20 ng/ml TGF-β1 increased the intensity of α-SMA more than 1.8-fold compared to the control, and intensity of F-actin increased 1.6-fold compared to the control (Fig. 4B). Expression of fibroblast marker, α-SMA was increased after TGF-β1 treatment in HUVEC cells (Fig. 4C). Next, we examined the effects of retinoic acid and forskolin on TGFβ1-induced HUVEC activation using the cellular phenotype-based 2D assay system. In this experiment, we found that 1 µM retinoic acid and forskolin decreased the expression of α-SMA after TGF-β1 treatment in HUVECs relative to treatment with 10 µM pirfenidone (Fig. 4D). Of interest, expression of α-SMA were inhibited when 1 µM retinoic acid and forskolin were added with TGF-β1 to HUVEC cells, (Fig. 4E,F). Retinoic acid also more efficiently induced reprogramming of HUVEC activated than forskolin. These results suggested that anti-fibrotic compounds, such as retinoic acid and forskolin, suppress the EndMT process in HUVECs.

### The combination of anti-cancer drugs and anti-fibrosis compounds improves responses by enhancing penetration of anti-cancer drugs

Liver cancer patients typically experience fibrosis, cirrhosis, and liver-related disease. As shown in Fig. 2A,F, spheroids showed loose compactness after treatment with anti-fibrosis compounds, and cell–cell tight junction interactions were also weak compared to controls. In our previous study^11^, we compared the efficacy of drug penetration by detecting the distribution of doxorubicin using fluorescence microscopy in HepG2 spheroids and HepG2-MCTS grown with LX2 or WI38 cells. In this study, we sought to determine whether the anti-fibrosis compounds may increase the penetration of anti-cancer drugs in MCTSs by decreasing cell–cell interactions. When the MCTSs that were treated with 5 µM retinoic acid or forskolin were treated with 10 µM doxorubicin for 8 h, the distribution of doxorubicin in MCTSs was highly increased relative to spheroids that were not treated with retinoic acid or forskolin (Fig. 5A). Indeed, doxorubicin only penetrated the periphery of MCTSs after treatment with 0.5% DMSO. This result was not surprising in light of the observed decreased compactness of MCTSs after treatment with anti-fibrosis compounds. Based on these results, we expected that anti-fibrosis compounds may accelerate anti-cancer effects by enabling delivery of anti-cancer compounds to the center of tumor tissues. In general, apoptosis-inducing mechanism were investigated refer to evaluate the anti-cancer effects. Among the apoptosis markers, caspase 3 play a role of collaborating the distribution of cellular structure including degradation of DNA and cytoskeleton proteins. In this study, we found that spheroids treated with 1 µM retinoic acid or forskolin combined with 1 or 3 µM of sorafenib had high expression of cleaved caspase-3, as an apoptosis marker, in MCTS model.

Caspase 3 significantly higher relative to spheroids treated with sorafenib alone (Fig. 5B). From these results, it appears that anti-fibrosis compounds, such as retinoic acid or forskolin, may improve the efficacy of anti-cancer drugs and attenuate tissue compactness and stiffness observed in liver fibrosis.

## Discussion

Fibrosis has been identified as a key factor that influences survival in patients with non-alcoholic steatohepatitis (NASH)^29,30^. Hepatic fibrosis frequently progresses to cirrhosis and hepatocellular carcinoma, but it does not cause symptoms itself. Since there is currently no standard treatment for hepatic fibrosis, there is currently a strong incentive for pharmaceutical companies to develop safe and effective therapeutics^29^. Further, because there are no therapies currently approved for the treatment of hepatic fibrosis, this disease is designated by the FDA as a Fast Track Development indication^31^. In recent years, strategies of target-based approaches for screening small-molecules have shifted the strategic landscape in the evaluation of drugs that may treat hepatic fibrosis^32^. FXR agonists (such as obeticholic acid) have demonstrated a dramatic reduction in progression and improvement in fibrosis in a phase 2 clinical study, but ultimately failed in phase 3 clinical study because of long-term toxicity^33^. ASK-1(MAP3 kinase 5) inhibition with selonsertib reduced hepatic fibrosis in mouse models, but phase 3 study of selonsertib failed to reprogram hepatic fibrosis^34^. Additionally, a C–C chemokine receptor type 2 (CCR2) and type 5 (CCR5) antagonist (cenicriviroc) provided anti-fibrotic activity in adult patients with hepatic fibrosis, but the anti-fibrotic effect did not meet the primary end point in a phase 2 clinical study. Hence, there remains a significant unmet need for safe and effective medications for the treatment of hepatic fibrosis in NASH^35^.

Investigations of the molecular mechanisms of hepatic fibrosis have presented several clear targets such as TGF-β1, PPAR, ASK-1, angiotensin, YAP-TEAD, various inflammatory cytokines, and ROS. However, there remains no validated target for novel anti-fibrotic compounds^36,37^.

To develop the novel compounds for hepatic fibrosis, we first need to understand the complexity of the molecular mechanisms that govern hepatic fibrogenesis and the local microenvironment. Hepatic fibrosis is caused by chronic inflammation, and the liver tissue becomes rigid due ECM accumulation. Further, this environment results in EMT or EndMT activation^38^. There have been numerous studies assessing novel drugs for fibrosis and molecular mechanisms using 2D culture systems for cells in monolayer on plastic culture dishes. However, recent evidence has suggested that 2D systems fail to capture several crucial elements of the 3D environment, and 3D culture systems may be a more effective culture method. Diverse phenotypic approaches for drug screening assays have become increasingly popular in drug discovery as an alternative strategy to target-based approaches for the assessment of potential treatments for hepatic fibrosis^39–41^. Particularly, 3D co-culture models represent a high-throughput phenotypic screening system to efficiently screen for new anti-fibrotic therapeutics^42–46^. In this study, we tested whether MCTS models may recapitulate the in vivo microenvironment in fibrosis to generate a phenotype-based model that could overcome the shortcomings seen with 2D systems.

In addition to abnormal HSC activation, ECM deposition and stiffness are key phenotypes observed in hepatic fibrosis in vivo^38,47^. Thus, strategies to reverse HSC activation, ECM deposition, and stiffness in spheroid models are critical to develop effective therapeutic agents for fibrosis. We found that MCTSs possessed ECM structural constituents (Fig. 1). Currently,the most common agents that are prescribed off-label for hepatic fibrosis in NASH include vitamin E, ursodeoxycholic acid, pioglitazone, metformin, and lipid-modifying agents^48,49^. Retinoic acid, retinoic acid analogs, and cAMP activators such as forskolin can attenuate hepatic stellate cell activation and have been validated with animal studies^50–53^. Interestingly, a series of off-label agents for hepatic fibrosis such as ursocholanic acid, rosiglitazone, and retinoic acid analogs were included among the HIT compounds identified herein (Fig. 2, Table 1). Forskolin and forskolin derivatives (NKH 477) attenuate carbon tetrachloride-induced liver fibrosis in rats^54^ and were also identified as HIT compounds. The anti-fibrotic effects of forskolin have already been shown in animal models of liver fibrosis^54,55^ and intestinal organoids^46^. Hence, our MCTS-based screening system appears to represent an effective approach for the identification of future therapeutics of fibrosis, providing comparable results with animal experiments.

TGF-β plays a key role in the progression of liver fibrosis, and drugs that inhibit TGF-β have been shown to have anti-fibrotic effects in animal studies^56^. The MCTS model used in this study also represented the ECM-related protein accumulation as well as p-smad activation (Fig. 1). We found that reducing the expression of CD133, a cancer stem cell marker associated with liver cancer, had anti-fibrotic effects and also regulates the surrounding environment, potentially influencing risk of HCC.

Moreover, when we developed spheroids using normal hepatocytes instead of HCC cells in MCHSs, they showed ECM accumulation or mesenchymal cell properties, but did not change the phenotypic properties after drug treatment. From this result, HCC cells with stromal cells appear to best represent pathological characteristics of hepatic fibrosis.

As mentioned earlier, 2D phenotypic assay systems have been adapted for selecting anti-fibrotic compounds through LX2 cell activation with TGF-β1. In Figs. 3 and 4, we also utilized this system for secondary validation of hit compounds from the MCTS-based screening. Hit compounds from the screen were also effective at inhibiting endothelial activity and hepatic stellate activation. The failure rate in clinical trials could possibly be reduced by deriving the first hit through MCTS-based screens and then verifying drug efficacy in a 2D assay that can verify inhibition of EMT and EndMT, followed by confirmation in animal models of disease.

Biopsies of tissues from HCC patients commonly show evidence of cirrhosis and NASH. It has been suggested that drug treatment efficacy in liver cancer patients is lower than other carcinomas due to the hepatic microenvironment^30^. Tissue rigidity due to the accumulation of ECM and excessive inflammatory reactions lowers drug permeability, which reduces the ability of therapeutic compounds to access target cells. We have previously reported that losartan reduced the robustness of MCTS and consequently increased the permeability to doxorubicin. Similarly, in this work, drug permeability was increased when the anti-fibrotic drugs retinoic acid and forskolin were used to treat MCTSs (Fig. 5). The anti-cancer effect of sorafenib, a common treatment for liver cancer, was improved after combined treatment with these anti-fibrotics identified in our model system.

In this study, an in vitro model that reflects the microenvironment observed in hepatic fibrosis in vivo was constructed, characterized, and tested as a model for screening drugs that may be effective treatments for liver fibrosis. We expect that this model offers an efficient, high-throughput strategy to identify new drugs and targets through phenotypic screening. We found that anti-fibrotic drugs are not only effective in the treatment of liver fibrosis, but can also enhance the anti-cancer activity of other therapeutics by increasing tissue permeability, allowing drug delivery to cancer cells of interest.

## Supplementary Information

Supplementary Figures.

## References

[1] Bataller R, Brenner DA (2005). Liver fibrosis. J. Clin. Investig..

[2] Weiskirchen, R., Weiskirchen, S. & Tacke, F. Recent advances in understanding liver fibrosis: Bridging basic science and individualized treatment concepts. *F1000Research* **7**. 10.12688/f1000research.14841.1 (2018). 10.12688/f1000research.14841.1 PMC6024236 30002817

[3] Woolbright BL, Jaeschke H (2018). Alcoholic hepatitis: Lost in translation. J. Clin. Transl. Hepatol..

[4] Kleiner DE (2017). Drug-induced liver injury: The hepatic pathologist's approach. Gastroenterol. Clin. N. Am..

[5] Penz-Osterreicher M, Osterreicher CH, Trauner M (2011). Fibrosis in autoimmune and cholestatic liver disease. Best Pract. Res. Clin. Gastroenterol..

[6] Brunt EM (2004). Nonalcoholic steatohepatitis. Semin. Liver Dis..

[7] Clark AM (2014). A microphysiological system model of therapy for liver micrometastases. Exp. Biol. Med..

[8] Kovacs EJ, DiPietro LA (1994). Fibrogenic cytokines and connective tissue production. FASEB J..

[9] Zhang CY, Yuan WG, He P, Lei JH, Wang CX (2016). Liver fibrosis and hepatic stellate cells: Etiology, pathological hallmarks and therapeutic targets. World J. Gastroenterol..

[10] Dufton NP (2017). Dynamic regulation of canonical TGFbeta signalling by endothelial transcription factor ERG protects from liver fibrogenesis. Nat. Commun..

[11] Song Y (2016). Activated hepatic stellate cells play pivotal roles in hepatocellular carcinoma cell chemoresistance and migration in multicellular tumor spheroids. Sci. Rep..

[12] Moreira RK (2007). Hepatic stellate cells and liver fibrosis. Arch. Pathol. Lab. Med..

[13] Pardali, E., Sanchez-Duffhues, G., Gomez-Puerto, M. C. & Ten Dijke, P. TGF-beta-induced endothelial-mesenchymal transition in fibrotic diseases. *Int.**J.**Mol.**Sci.* **18**. 10.3390/ijms18102157 (2017). 10.3390/ijms18102157 PMC5666838 29039786

[14] Kim SH, Song Y, Seo HR (2019). GSK-3beta regulates the endothelial-to-mesenchymal transition via reciprocal crosstalk between NSCLC cells and HUVECs in multicellular tumor spheroid models. J. Exp. Clin. Cancer Res. (CR).

[15] Song Y (2017). TGF-beta-independent CTGF induction regulates cell adhesion mediated drug resistance by increasing collagen I in HCC. Oncotarget.

[16] Minchinton AI, Tannock IF (2006). Drug penetration in solid tumours. Nat. Rev. Cancer.

[17] Hirschhaeuser F (2010). Multicellular tumor spheroids: An underestimated tool is catching up again. J. Biotechnol..

[18] Jang JW (2016). Hepatocellular carcinoma-targeted drug discovery through image-based phenotypic screening in co-cultures of HCC cells with hepatocytes. BMC Cancer.

[19] Song Y, Kim IK, Choi I, Kim SH, Seo HR (2018). Oxytetracycline have the therapeutic efficiency in CD133(+) HCC population through suppression CD133 expression by decreasing of protein stability of CD133. Sci. Rep..

[20] Chen YS (2011). CD133/Src axis mediates tumor initiating property and epithelial-mesenchymal transition of head and neck cancer. PLoS ONE.

[21] Liu K, Hao M, Ouyang Y, Zheng J, Chen D (2017). CD133(+) cancer stem cells promoted by VEGF accelerate the recurrence of hepatocellular carcinoma. Sci. Rep..

[22] Ding Q (2014). CD133 facilitates epithelial-mesenchymal transition through interaction with the ERK pathway in pancreatic cancer metastasis. Mol. Cancer.

[23] Hautekeete ML, Geerts A (1997). The hepatic stellate (Ito) cell: Its role in human liver disease. Virchows Arch..

[24] Venturi C (2016). Relevance of activated hepatic stellate cells in predicting the development of pediatric liver allograft fibrosis. Liver Transplant..

[25] Schaefer CJ, Ruhrmund DW, Pan L, Seiwert SD, Kossen K (2011). Antifibrotic activities of pirfenidone in animal models. Eur. Respir. Rev..

[26] Garcia L (2002). Pirfenidone effectively reverses experimental liver fibrosis. J. Hepatol..

[27] Lin X (2018). Nintedanib inhibits TGF-beta-induced myofibroblast transdifferentiation in human Tenon's fibroblasts. Mol. Vis..

[28] Song Y (2019). Identification of radiation-induced EndMT inhibitors through cell-based phenomic screening. FEBS Open Bio.

[29] Sircana, A., Paschetta, E., Saba, F., Molinaro, F. & Musso, G. Recent insight into the role of fibrosis in nonalcoholic steatohepatitis-related hepatocellular carcinoma. *Int.**J.**Mol.**Sci*. **20**. 10.3390/ijms20071745 (2019). 10.3390/ijms20071745 PMC6480228 30970564

[30] Taylor, R. S. *et**al*. Association between fibrosis stage and outcomes of patients with nonalcoholic fatty liver disease: A systematic review and meta-analysis. *Gastroenterology* **158**, 1611–1625 e1612. 10.1053/j.gastro.2020.01.043 (2020). 10.1053/j.gastro.2020.01.043 32027911

[31] Filozof C, Goldstein BJ, Williams RN, Sanyal A (2015). Non-alcoholic steatohepatitis: limited available treatment options but promising drugs in development and recent progress towards a regulatory approval pathway. Drugs.

[32] Popov Y, Schuppan D (2009). Targeting liver fibrosis: strategies for development and validation of antifibrotic therapies. Hepatology.

[33] Abenavoli, L., Falalyeyeva, T., Boccuto, L., Tsyryuk, O. & Kobyliak, N. Obeticholic acid: A new era in the treatment of nonalcoholic fatty liver disease. *Pharmaceuticals**(Basel)* **11**. 10.3390/ph11040104 (2018). 10.3390/ph11040104 PMC6315965 30314377

[34] Loomba R (2018). The ASK1 inhibitor selonsertib in patients with nonalcoholic steatohepatitis: A randomized, phase 2 trial. Hepatology.

[35] Lefere S, Devisscher L, Tacke F (2020). Targeting CCR2/5 in the treatment of nonalcoholic steatohepatitis (NASH) and fibrosis: Opportunities and challenges. Expert Opin. Investig. Drugs.

[36] Rockey, D. C. Current and future anti-fibrotic therapies for chronic liver disease. *Clin.**Liver**Dis.* **12**, 939–962, xi. 10.1016/j.cld.2008.07.011 (2008). 10.1016/j.cld.2008.07.011 PMC2610449 18984475

[37] Chang Y, Li H (2020). Hepatic antifibrotic pharmacotherapy: Are we approaching success?. J. Clin. Transl. Hepatol..

[38] Wells, R. G. Cellular sources of extracellular matrix in hepatic fibrosis. Clin. Liver Dis. **12**, 759–768, viii. 10.1016/j.cld.2008.07.008 (2008). 10.1016/j.cld.2008.07.008 PMC2617723 18984465

[39] Rehman, M. *et**al*. High-throughput screening discovers antifibrotic properties of haloperidol by hindering myofibroblast activation. *JCI**insight* **4**. 10.1172/jci.insight.123987 (2019). 10.1172/jci.insight.123987 PMC6538355 30996132

[40] Bollong MJ (2017). Small molecule-mediated inhibition of myofibroblast transdifferentiation for the treatment of fibrosis. Proc. Natl. Acad. Sci. U.S.A..

[41] Wang XT (2018). Establishing a cell-based high-content screening assay for TCM compounds with anti-renal fibrosis effects. Evid.-Based Complement. Altern. Med. (eCAM).

[42] Langhans SA (2018). Three-dimensional *in vitro* cell culture models in drug discovery and drug repositioning. Front. Pharmacol..

[43] Arai K (2016). A novel high-throughput 3D screening system for EMT inhibitors: A pilot screening discovered the EMT inhibitory activity of CDK2 inhibitor SU9516. PLoS ONE.

[44] Wenzel C, Otto S, Prechtl S, Parczyk K, Steigemann P (2015). A novel 3D high-content assay identifies compounds that prevent fibroblast invasion into tissue surrogates. Exp. Cell Res..

[45] Xu Q, Norman JT, Shrivastav S, Lucio-Cazana J, Kopp JB (2007). *In vitro* models of TGF-beta-induced fibrosis suitable for high-throughput screening of antifibrotic agents. Am. J. Physiol. Renal Physiol..

[46] Boj SF (2017). Forskolin-induced swelling in intestinal organoids: an *in vitro* assay for assessing drug response in cystic fibrosis patients. J. Vis. Exp. (JoVE).

[47] Park S (2018). GOLGA2 loss causes fibrosis with autophagy in the mouse lung and liver. Biochem. Biophys. Res. Commun..

[48] Eshraghian A (2017). Current and emerging pharmacological therapy for non-alcoholic fatty liver disease. World J. Gastroenterol..

[49] Dajani A, AbuHammour A (2016). Treatment of nonalcoholic fatty liver disease: Where do we stand? An overview. Saudi J. Gastroenterol..

[50] Shimizu H, Tsubota T, Kanki K, Shiota G (2018). All-trans retinoic acid ameliorates hepatic stellate cell activation via suppression of thioredoxin interacting protein expression. J. Cell. Physiol..

[51] Senoo, H. & Wake, K. Suppression of experimental hepatic fibrosis by administration of vitamin A. *Lab.**Invest.**J.**Technic.**Methods**Pathol.* **52**, 182–194 (1985). 2578584

[52] Parkes JG, Templeton DM (2003). Effects of retinol and hepatocyte-conditioned medium on cultured rat hepatic stellate cells. Ann. Clin. Lab. Sci..

[53] Murakami K (2011). Therapeutic effects of vitamin A on experimental cholestatic rats with hepatic fibrosis. Pediatr. Surg. Int..

[54] El-Agroudy NN, El-Naga RN, El-Razeq RA, El-Demerdash E (2016). Forskolin, a hedgehog signalling inhibitor, attenuates carbon tetrachloride-induced liver fibrosis in rats. Br. J. Pharmacol..

[55] Pinzani M, Rombouts K, Colagrande S (2005). Fibrosis in chronic liver diseases: Diagnosis and management. J. Hepatol..

[56] Dewidar, B., Meyer, C., Dooley, S. & Meindl-Beinker, A. N. TGF-beta in hepatic stellate cell activation and liver fibrogenesis-updated 2019. *Cells* **8**. 10.3390/cells8111419 (2019). 10.3390/cells8111419 PMC6912224 31718044

