# Supplementary material for: Identification of hepatic fibrosis inhibitors through morphometry analysis of a hepatic multicellular spheroids model
Source: Sci Rep. 2021 May 25;11:10931. doi: 10.1038/s41598-021-90263-x (PMC8149639; doi:10.1038/s41598-021-90263-x)

Identification of hepatic fibrosis inhibitors through morphometry analysis of hepatic multicellular spheroids models

Yeonhwa Song[1§], Sanghwa Kim[1§], Jinyeong Heo[2], David Shum[2], Su-Yeon Lee[1], Minji Lee[1,3], A-Ram Kim[1] and Haeng Ran Seo[1*]

Author Affiliations:

[1] Cancer Biology Laboratory, [2] Screening Discovery Platform, Institut Pasteur Korea, 16, Daewangpangyo-ro 712 beon-gil, Bundang-gu, Seongnam-si, Gyeonggi-do, 13488, Korea, [3] Division of Bio-Medical Science & Technology, University of Science and Technology, Deajeon, 34113, Republic of Korea

[§]These authors contributed equally to this work.

*Corresponding author: Haengran Seo, Cancer Biology Research Laboratory, Institut Pasteur Korea, 16, Daewangpangyo-ro 712 beon-gil, Bundang-gu, Seongnam-si, Gyeonggi-do, 13488 Republic of Korea; Tel: +82-31-8018-8300; E-mail: shr1261@ip-korea.org

Supplementary Figure 1.

EndMT-, mesenchymal-, ECM-, TGFβ1 signaling-related protein expression levels in Spheroid and MCTS.

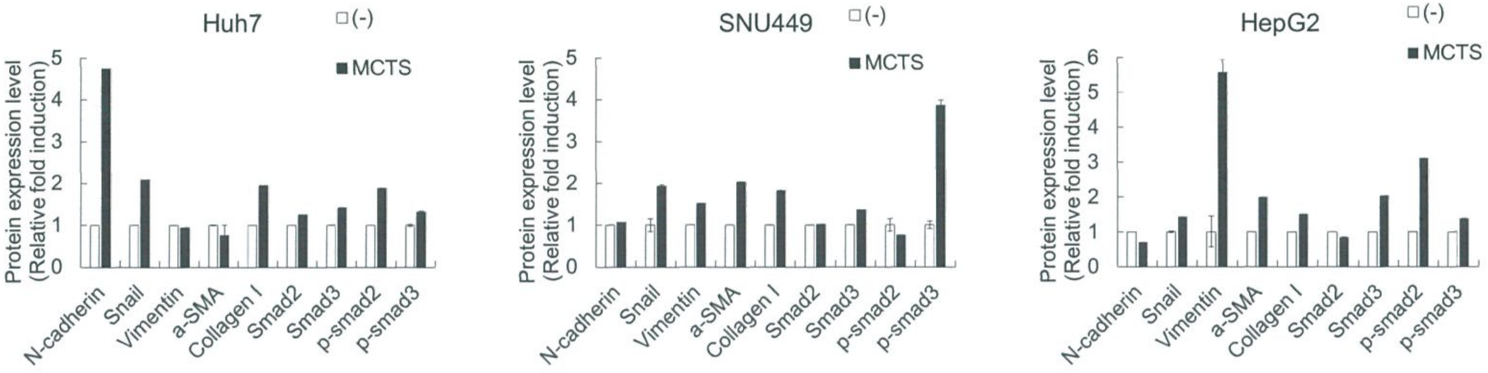

Supplementary Figure 2.
MCTS treated with 10μM Nintendnib, Pirfenidone, or Sorafenib.

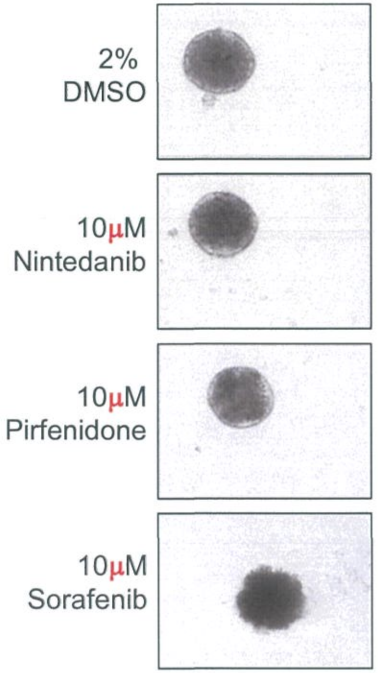

Supplementary Figure 3.

MCTS treated with hit compounds from MCTS-based phenomic screening. 0.1, 0.5, or 1μM of AM580, TTNPB, NHK477, and Iresogladine maleate were treated in MCTS.

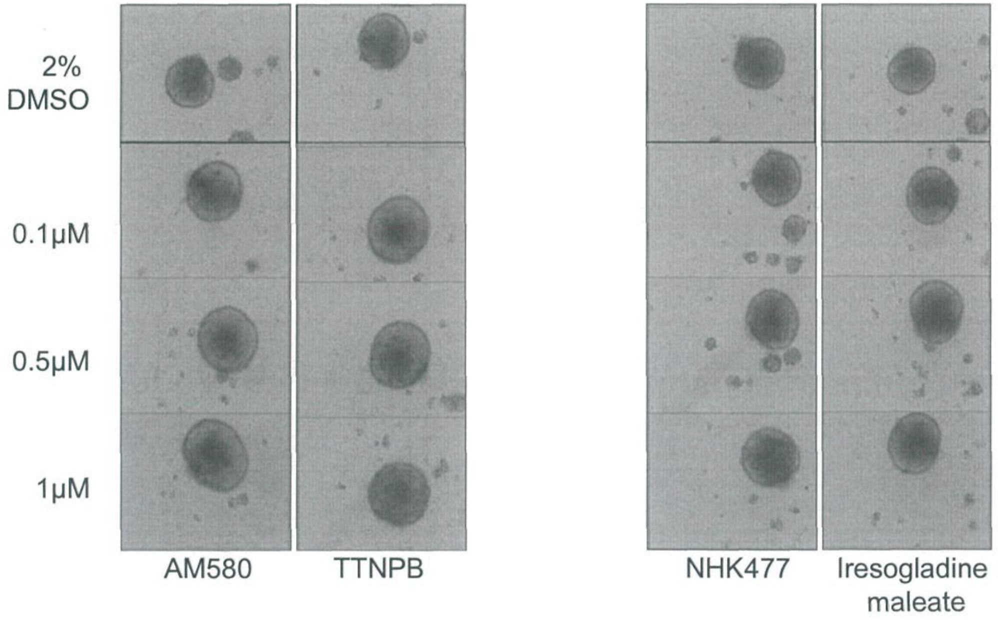

Supplementary Figure 4.

Expression of α-SMA and CD31 in tumor spheroids or MCTSs with or without treatment with 1 μM retinoic acid or forskolin.

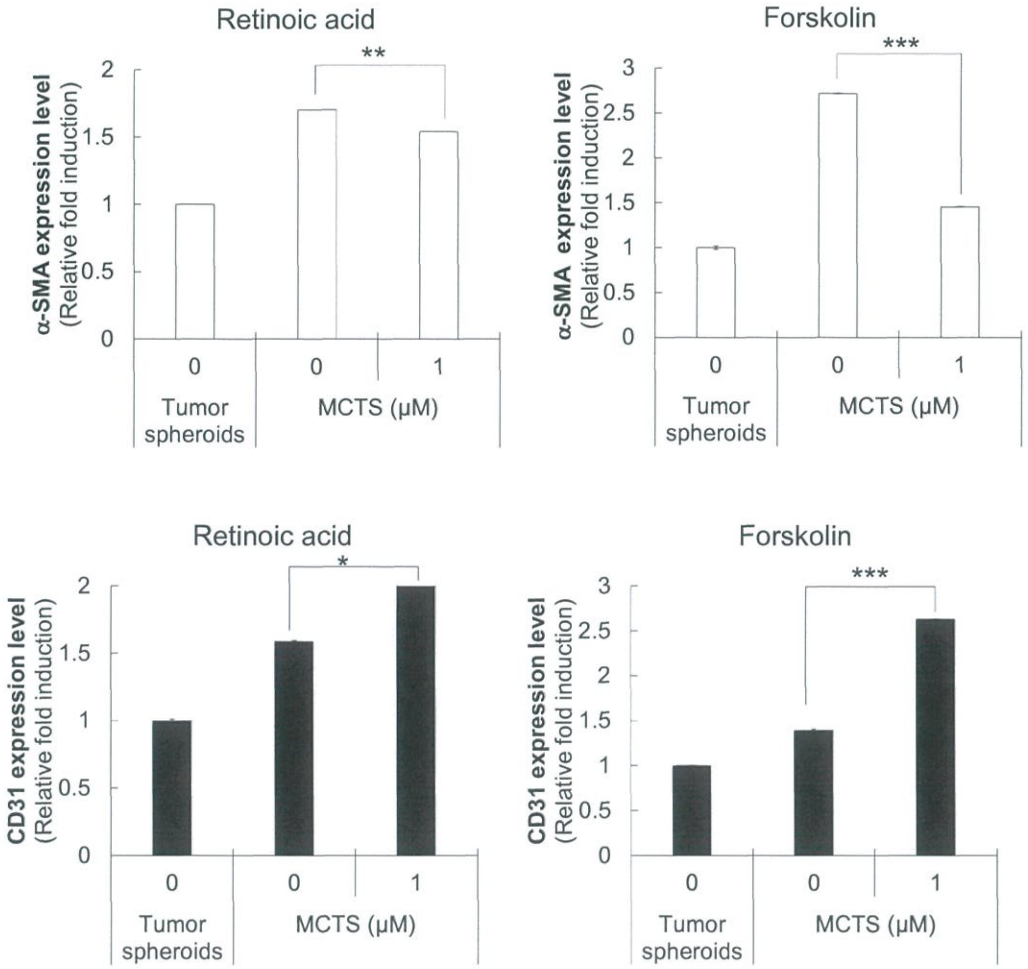

Supplementary Figure 5.

Expression of mesenchymal-related, endothelial-related, or cancer stem cell-related marker in hit compounds treated MCTS. α-SMA, which is mesenchymal-related marker, CD31, which is endothelial-related marker (A), CD133, which is cancer stem cell-related marker (B) were detected in 1μM of hit compounds, AM580, TTMPB, or NHK477, treated MCTS.

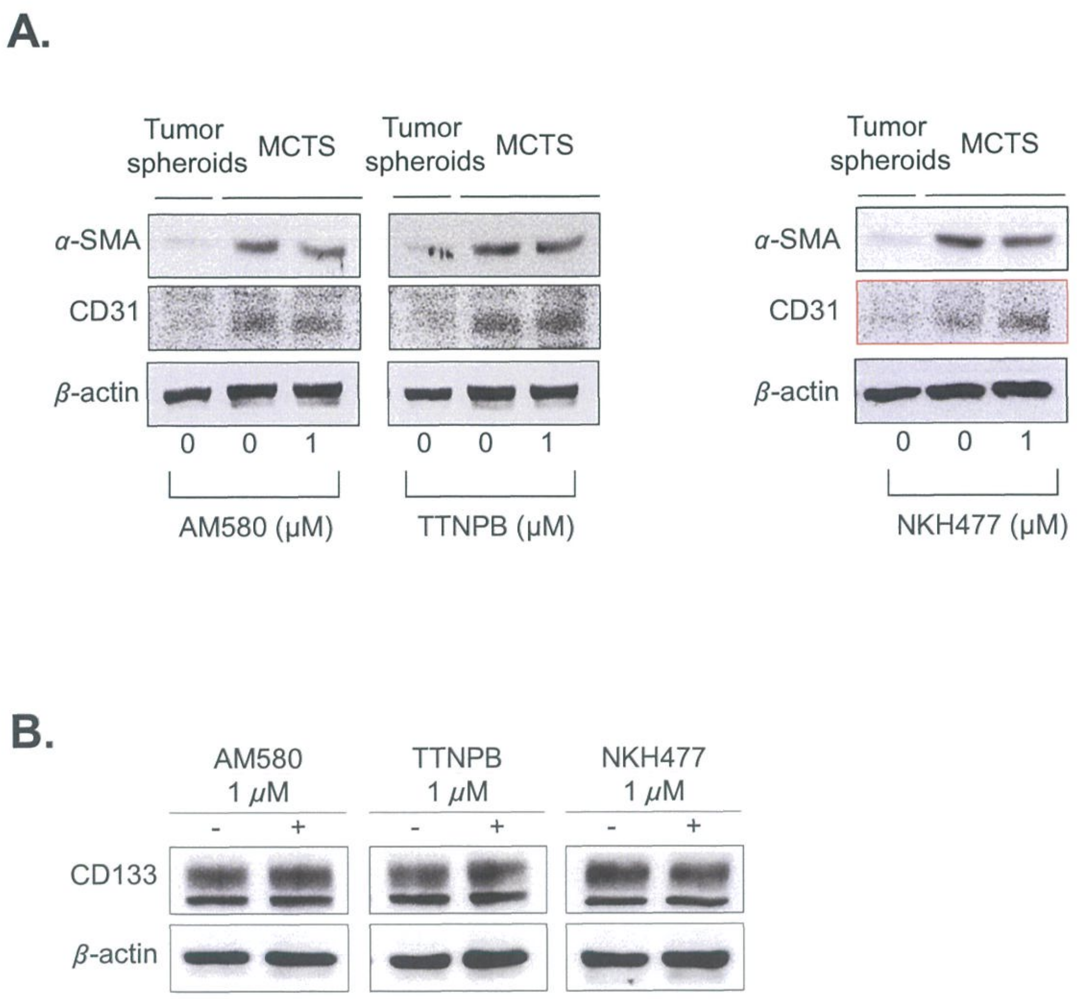

Supplementary Figure 6.

Expression of EMT-, mesenchymal-, EndMT-, ECM-related protein expression in hepatocyte spheroids and MCHSs.

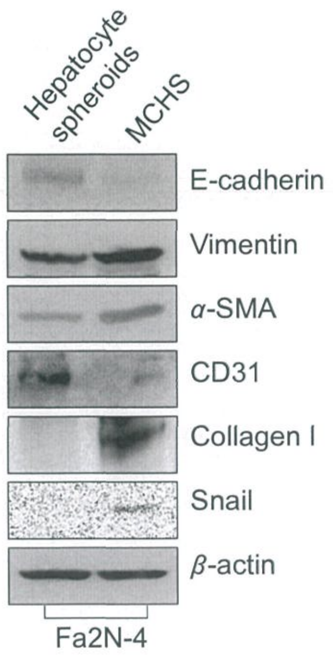

Supplementary Figure 7.

Expression of fibroblast-related markers including α-SMA. FAP, Collagen I in LX2 cell with treatment of TGFβ1

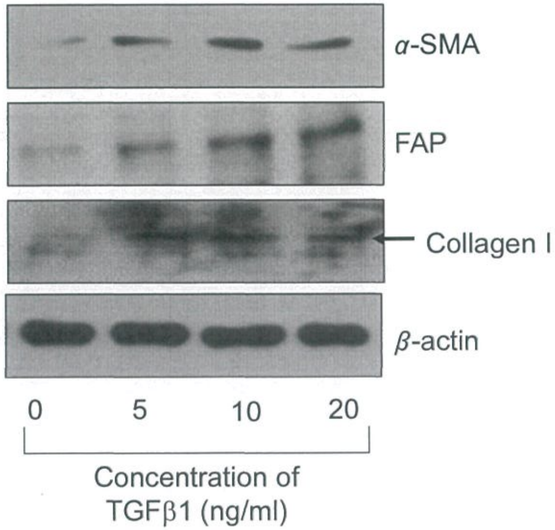

Supplementary Figure 8.

Intensity of α-SMA or F-actin in phenotypic-based 2D assay system using LX2 with 20ng/ml TGFβ1 and pirfenidone or nintedanib depending on the concentration from 20uM with 2-fold dilution.

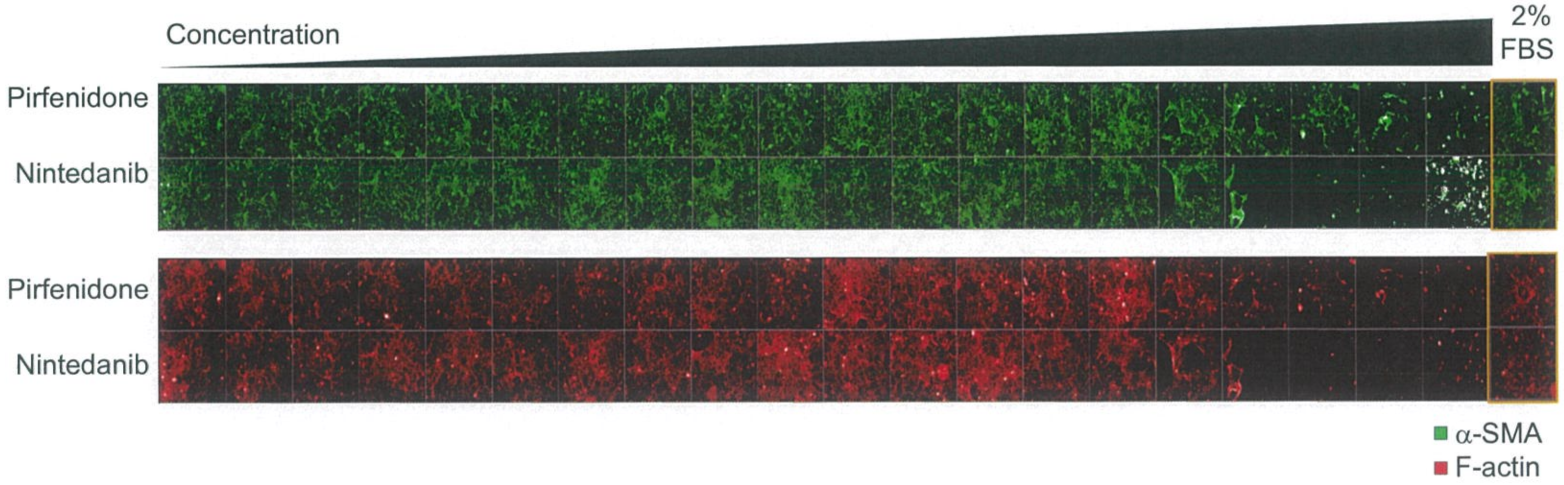

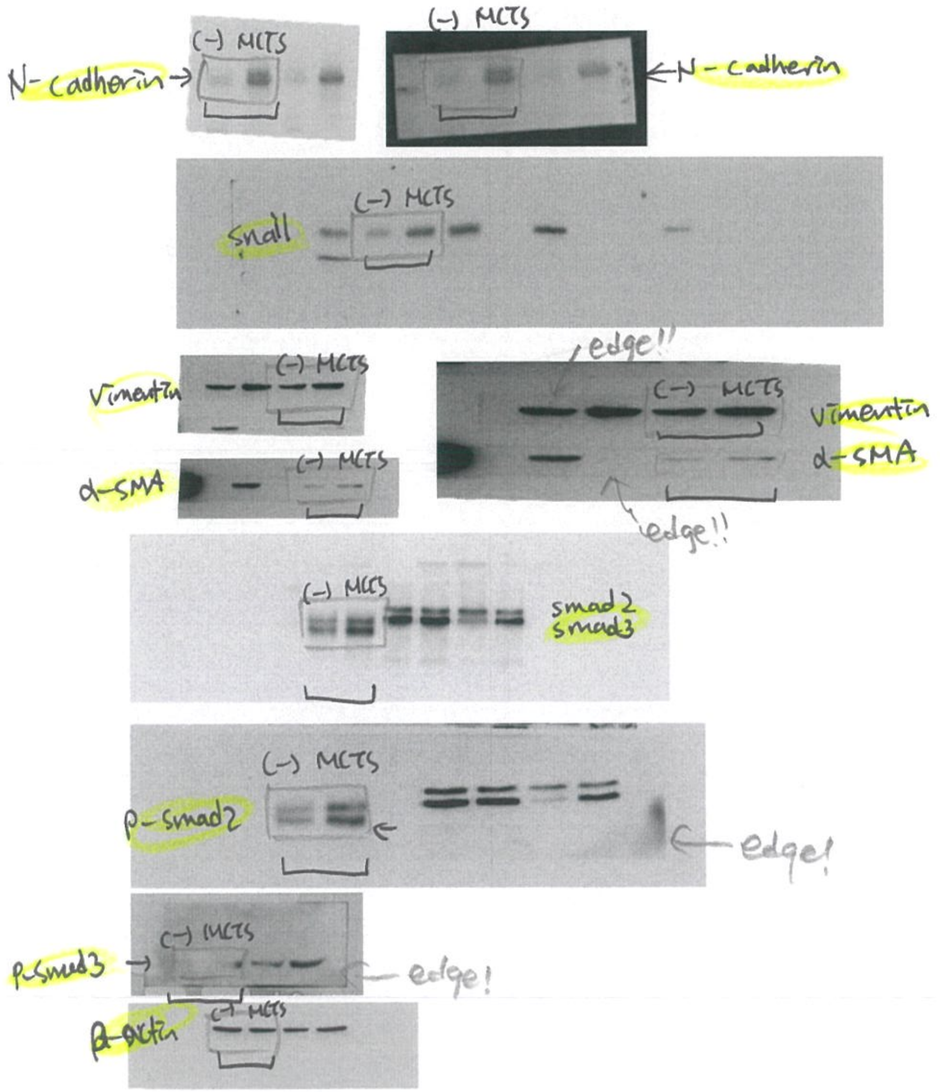

Figure 1C (Huh7)

Figure 1C (HepG2)

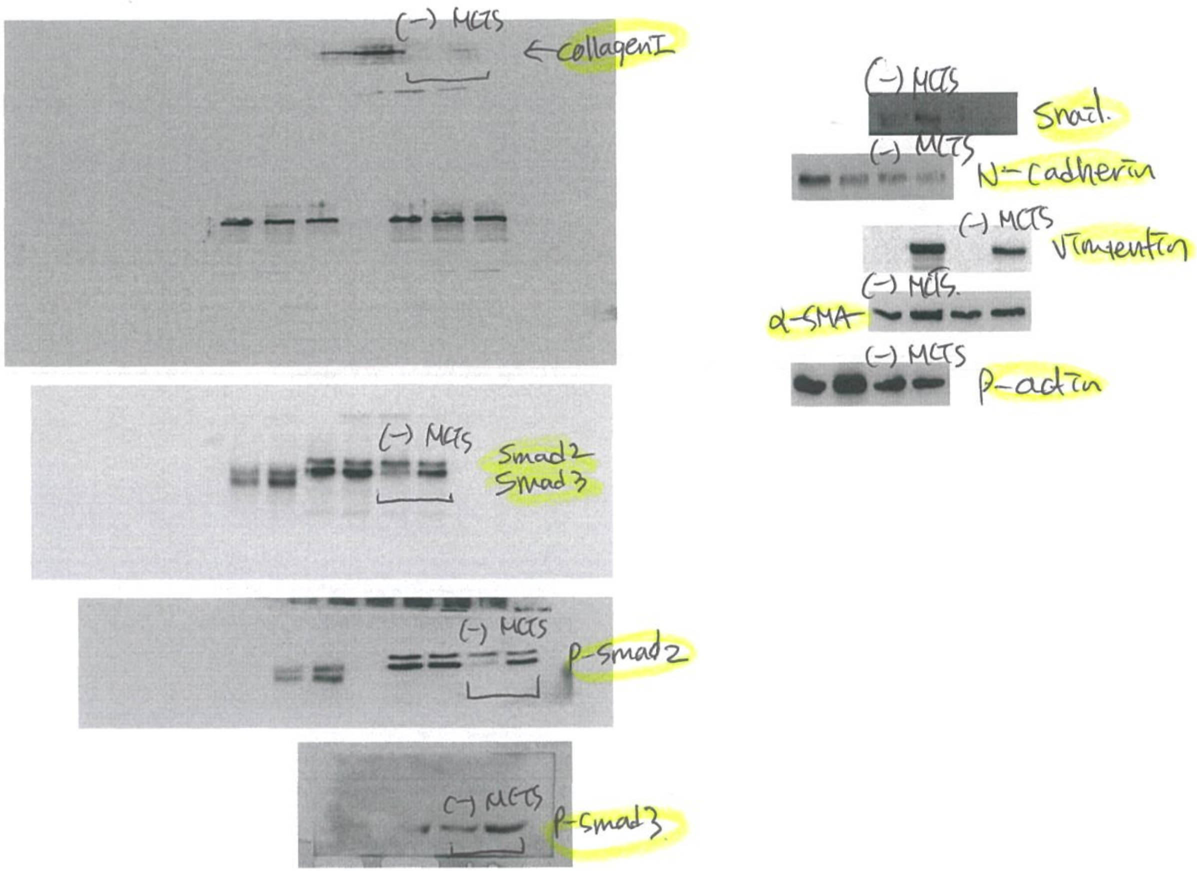

Figure 1C (SNU449)

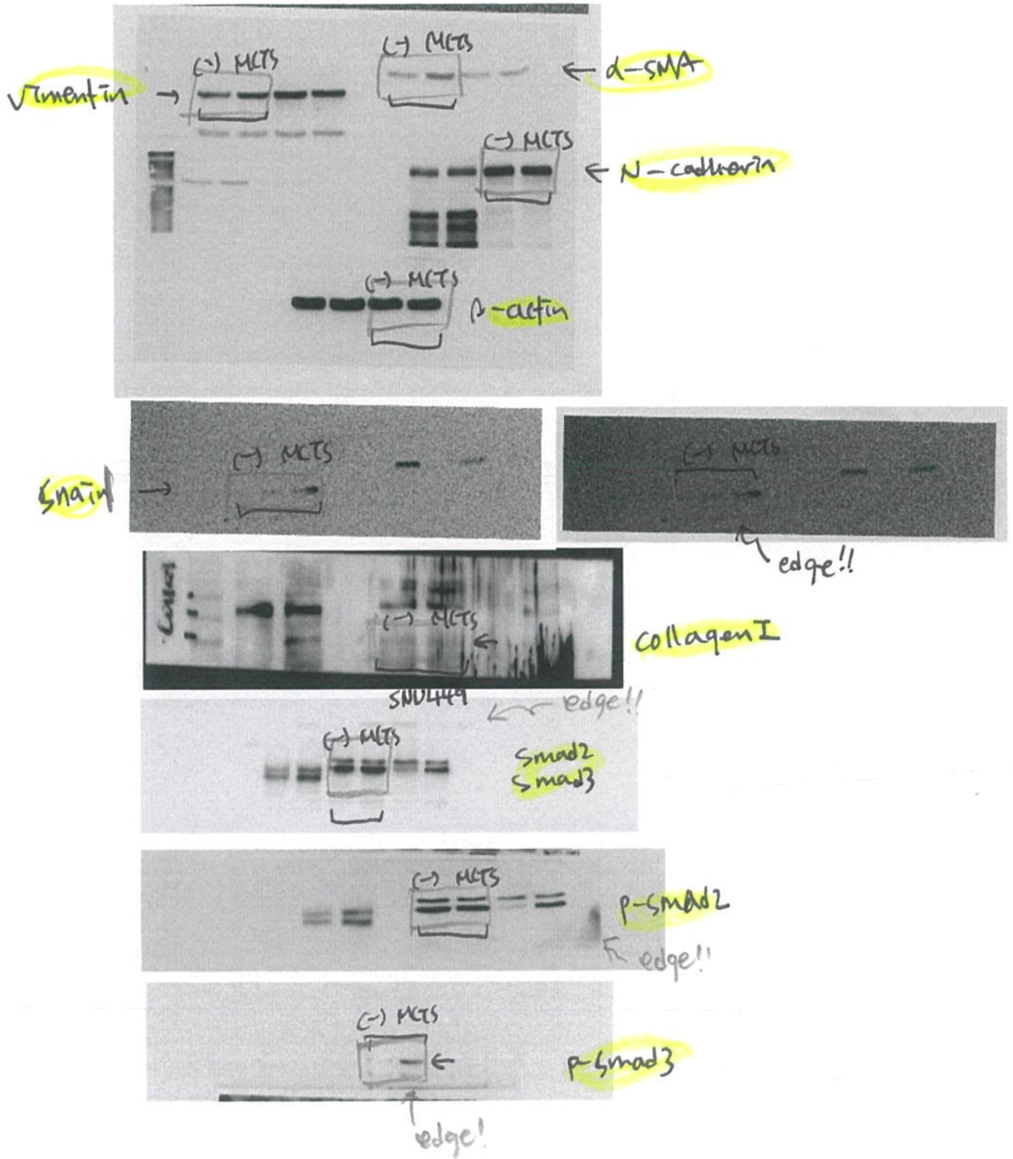

*T·S: Tumor spheroid

Figure 2B

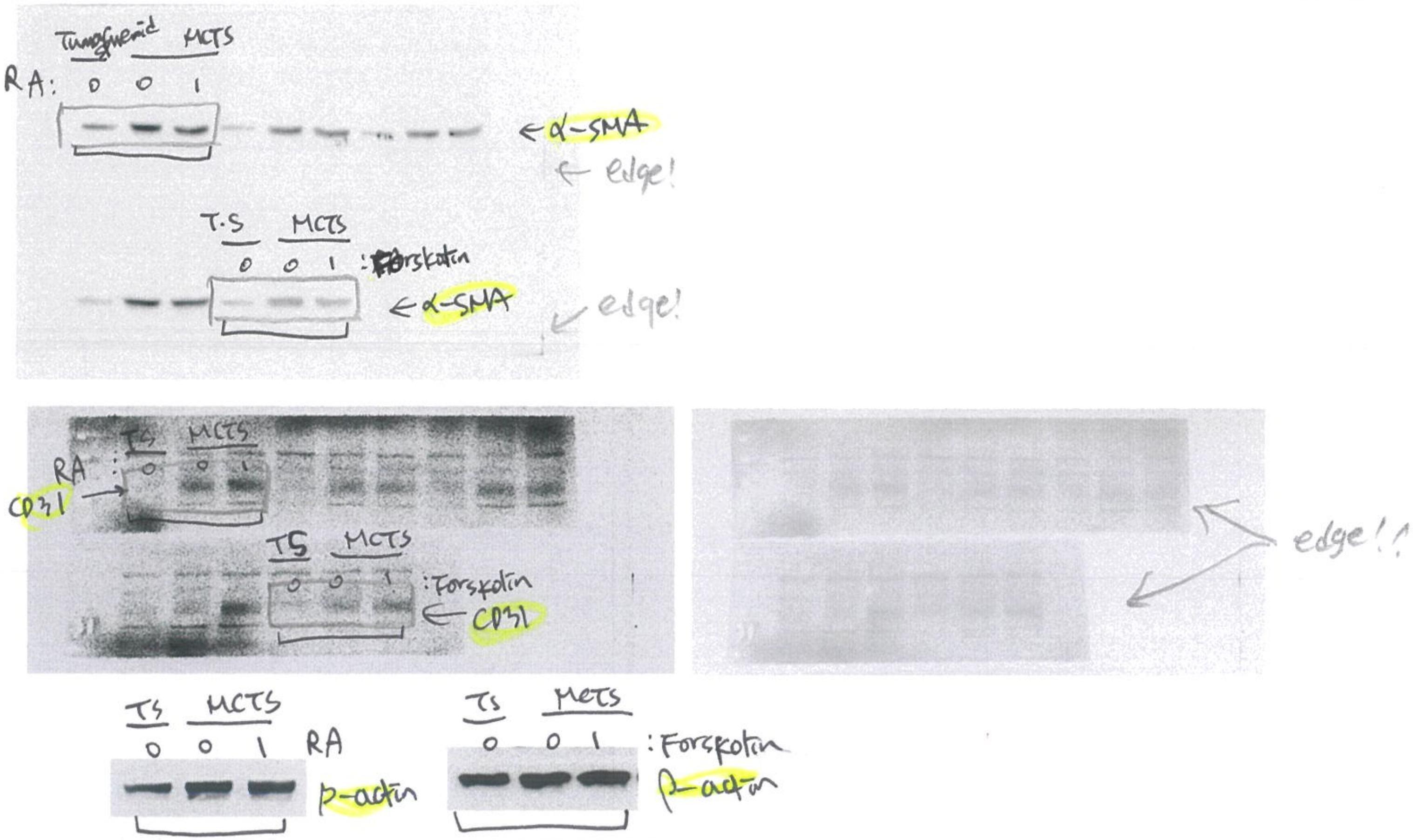

Figure 2C

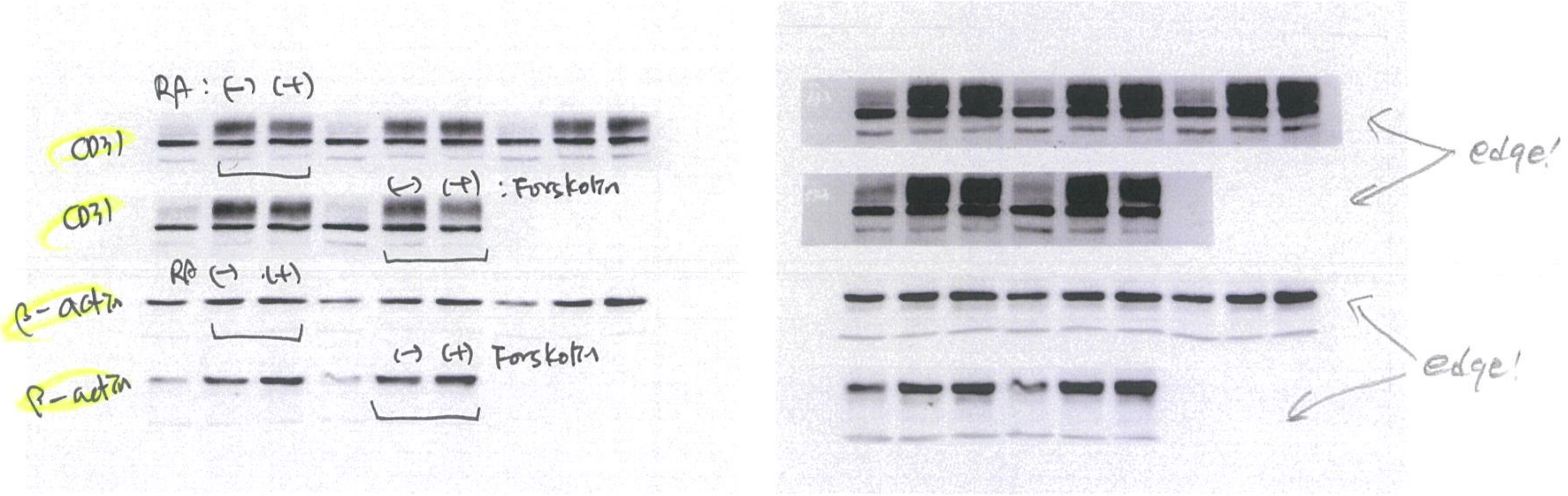
RA : (-) (+)
CD31
(-) (+) : Forskolin
CD31
RA (-) (+)
β-actin
(-) (+) Forskolin
β-actin
edge!
edge!

Figure 3E

R.A: Retinoic acid

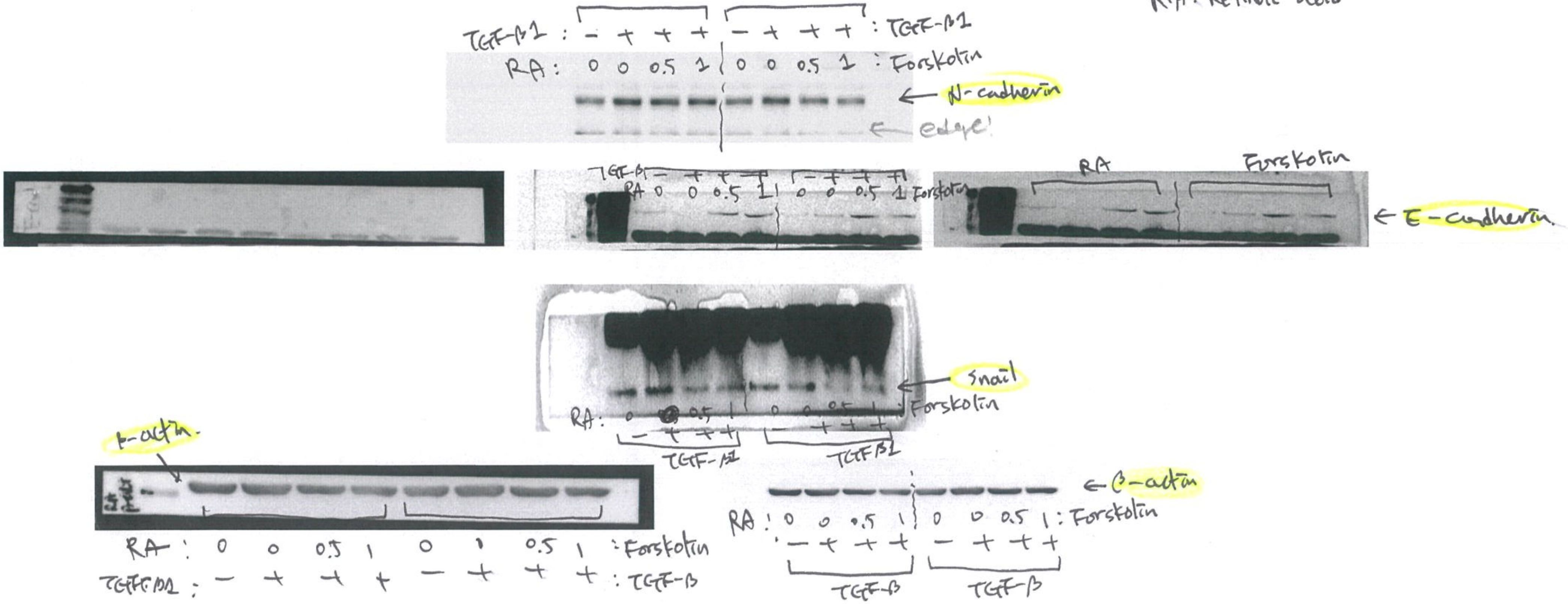

Figure 4C

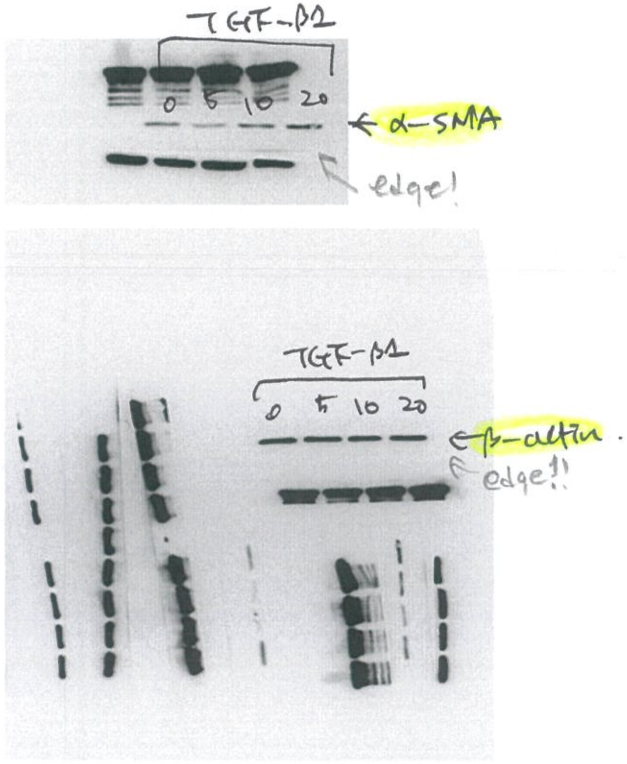

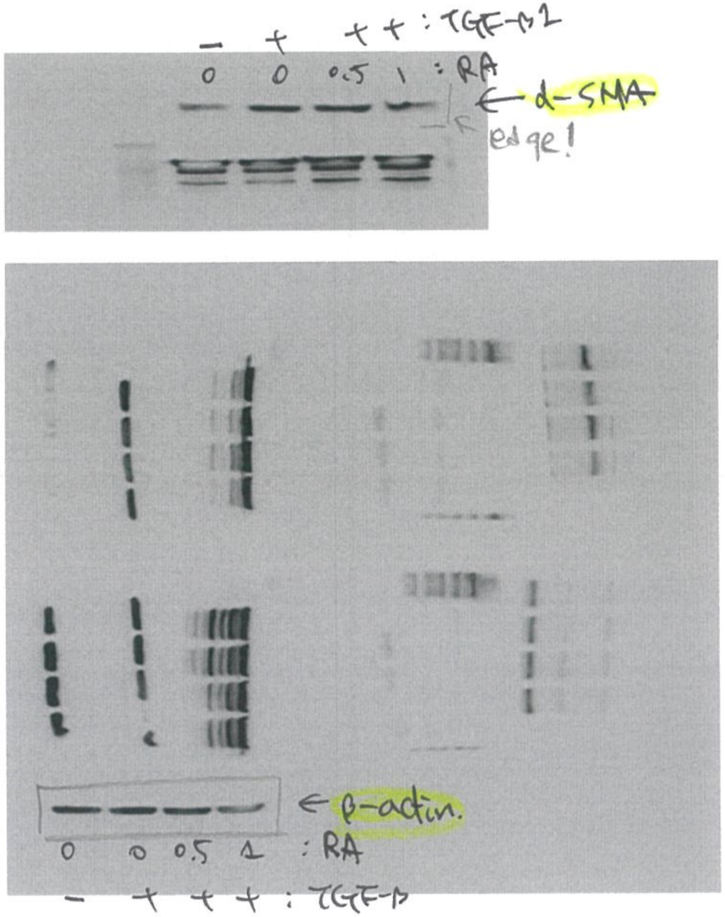

Figure 4E

*RA: Retinoic acid.

Figure 4F

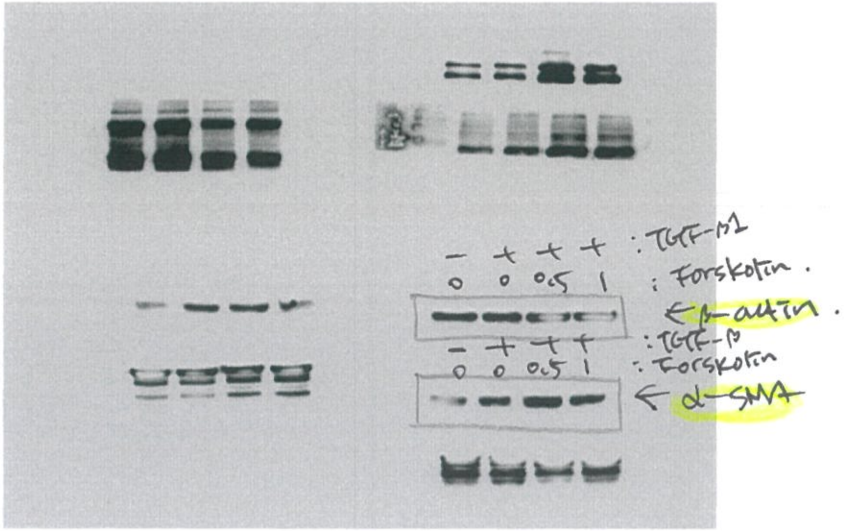

Figure 5B

*RA: Retinoic acid

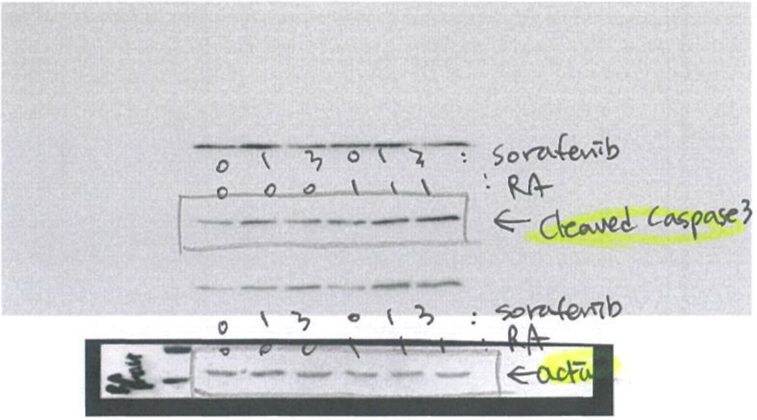

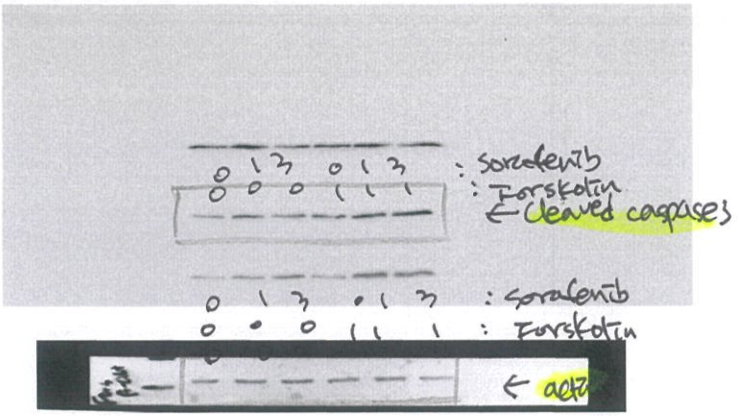

Supplementary Figure 1
(Huh7)

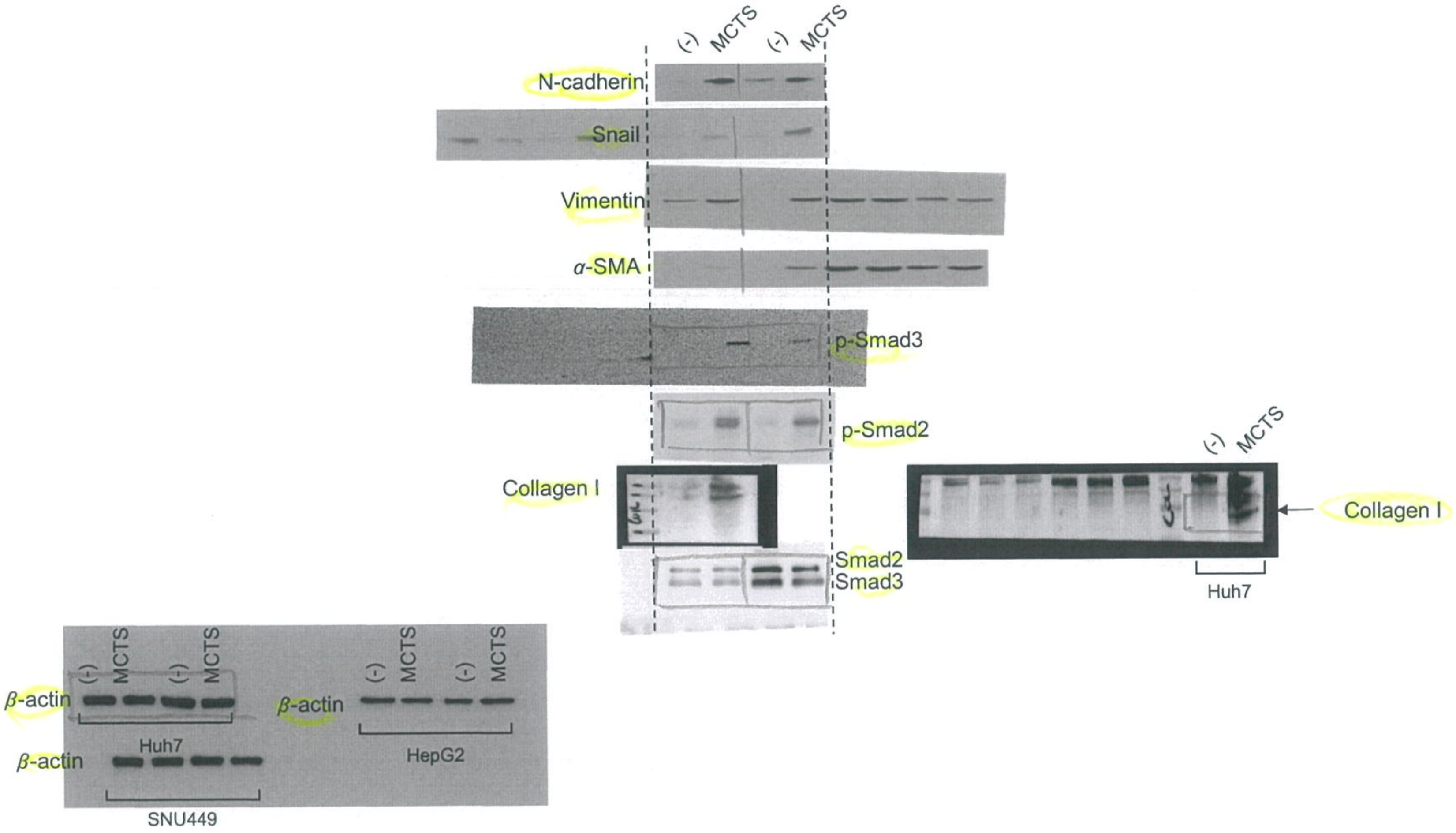

Supplementary Figure 1
(HepG2)

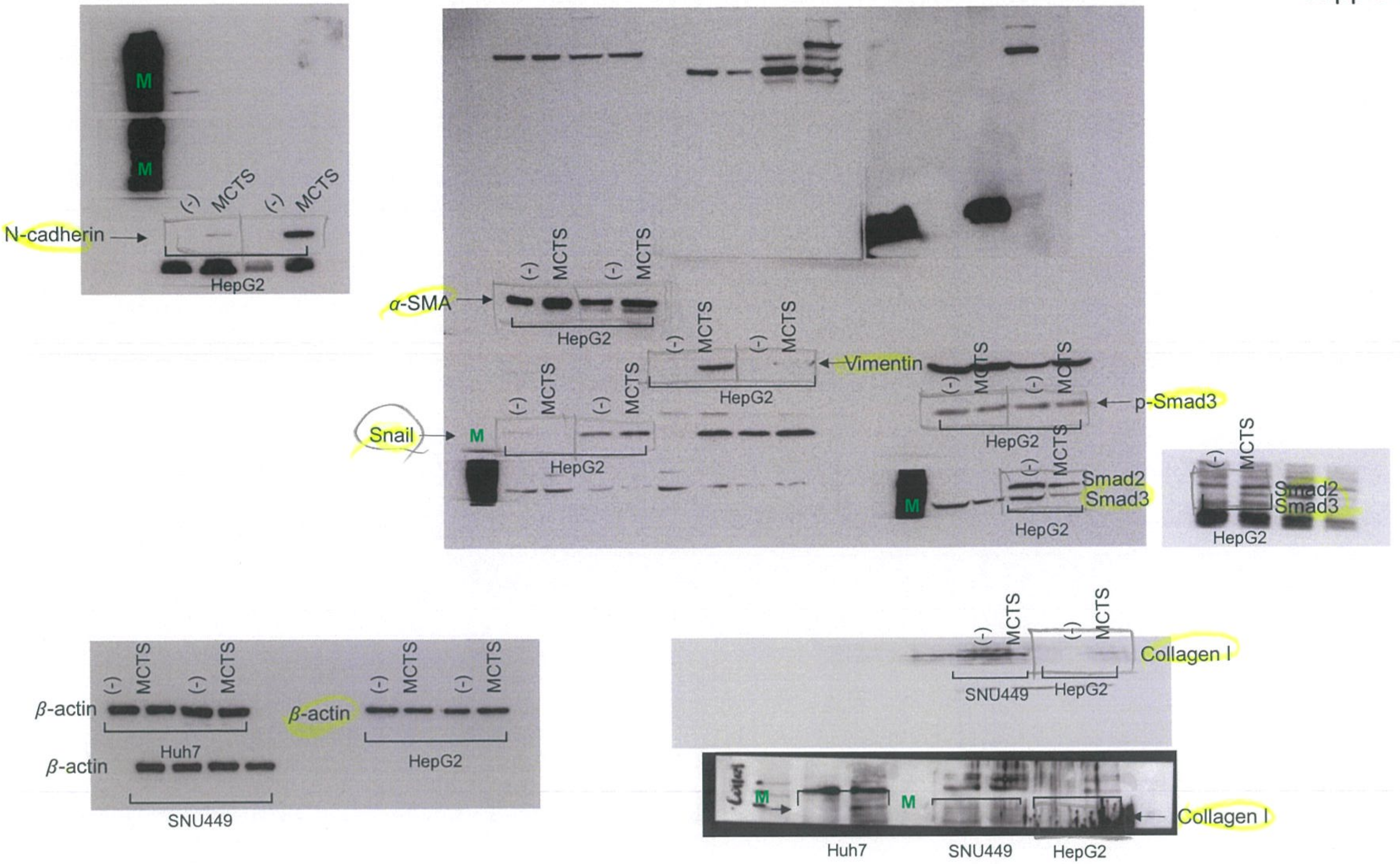

Supplementary Figure 1
(SNU449)

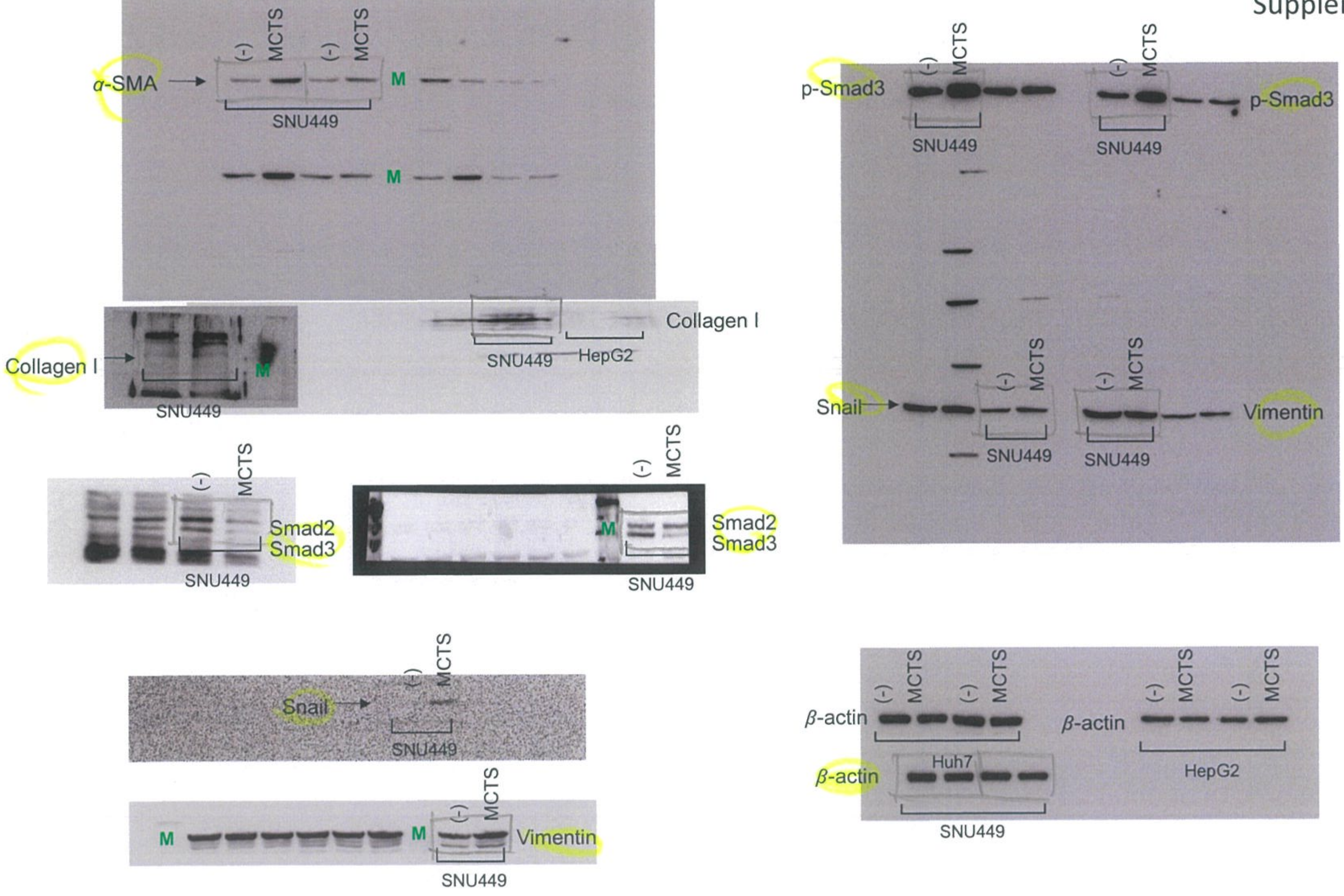

Supplementary Figure 4

Fig2B

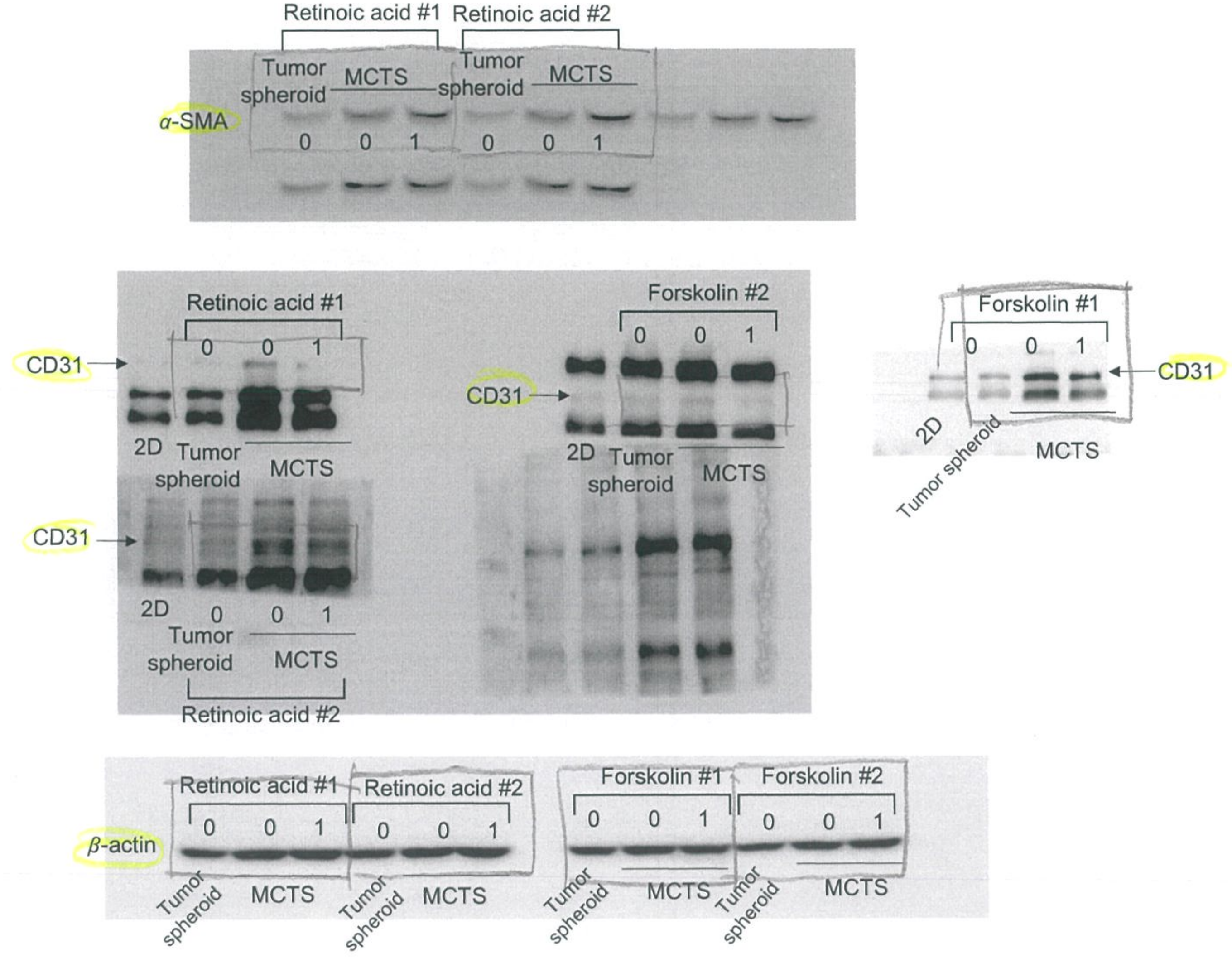

Supplementary figure 5A

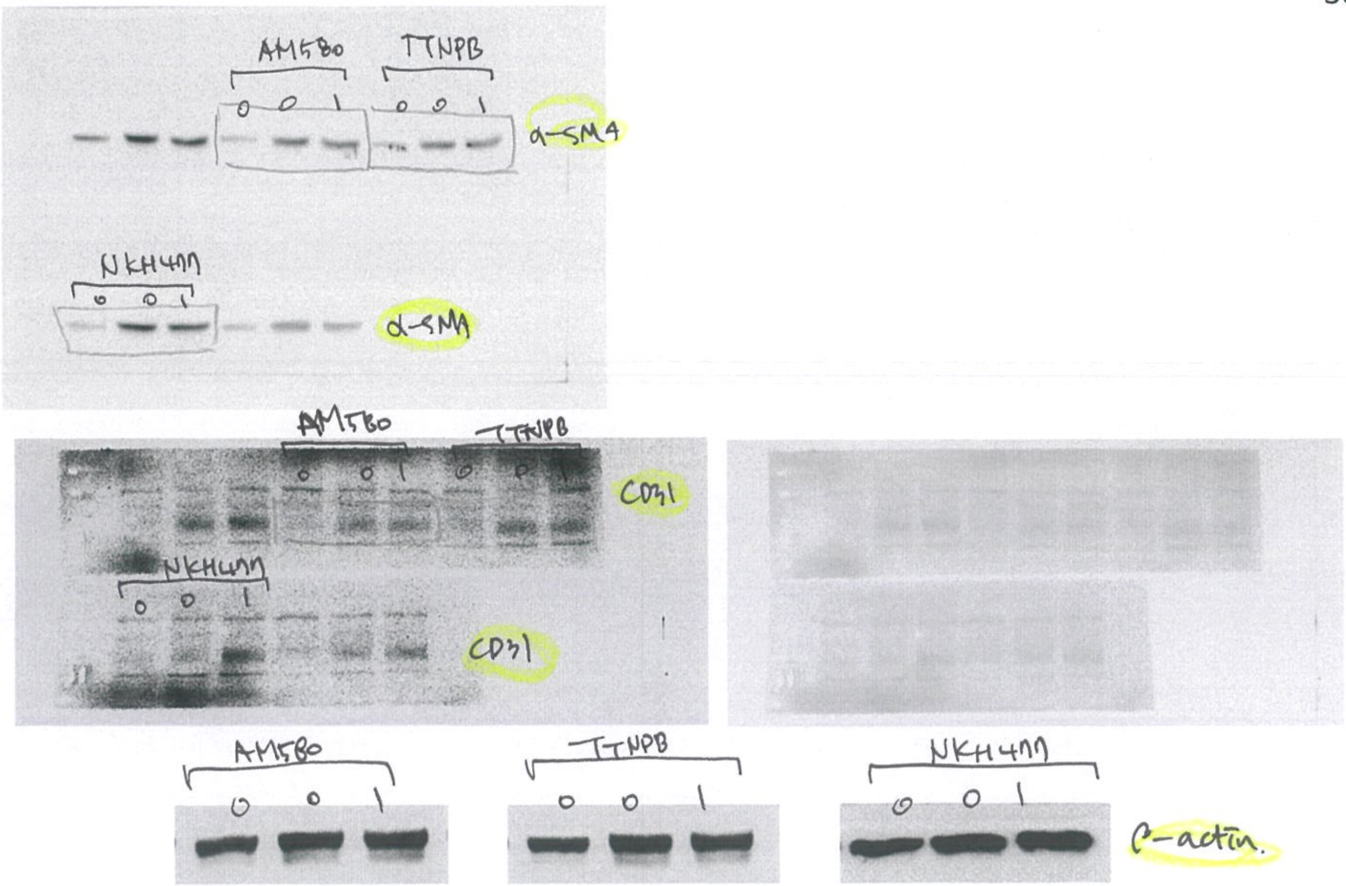

Supplementary figure 5B

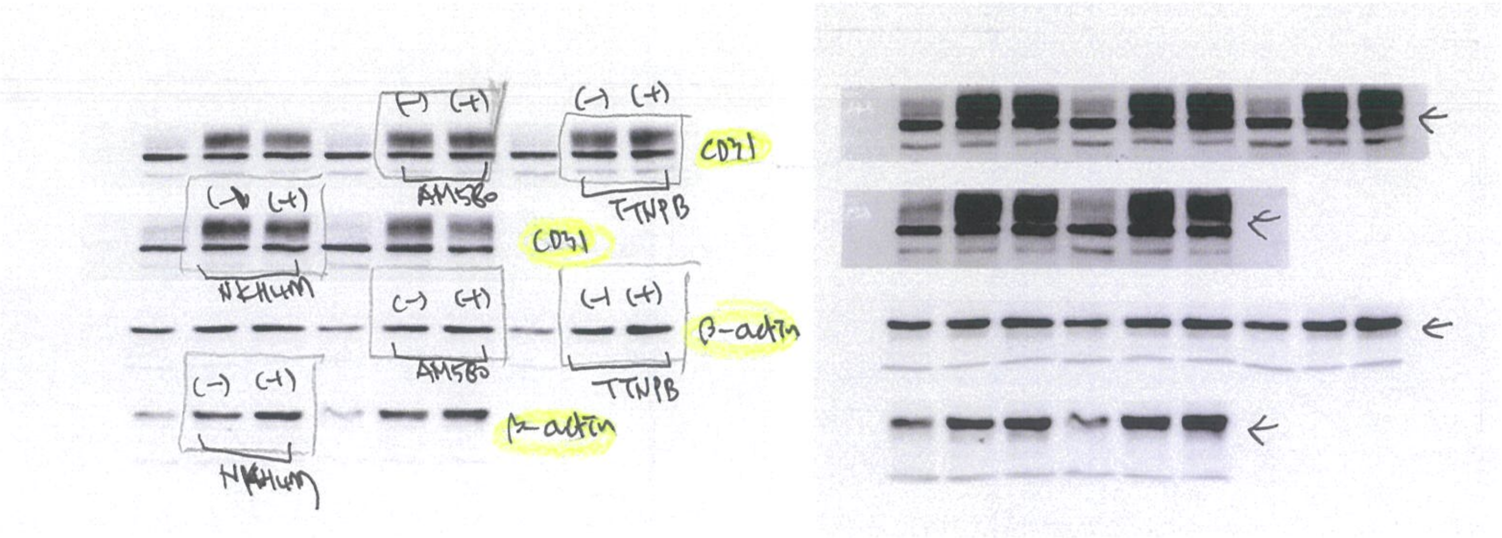

Supplementary figure 6

*H.S: Hepatocyte Speroid

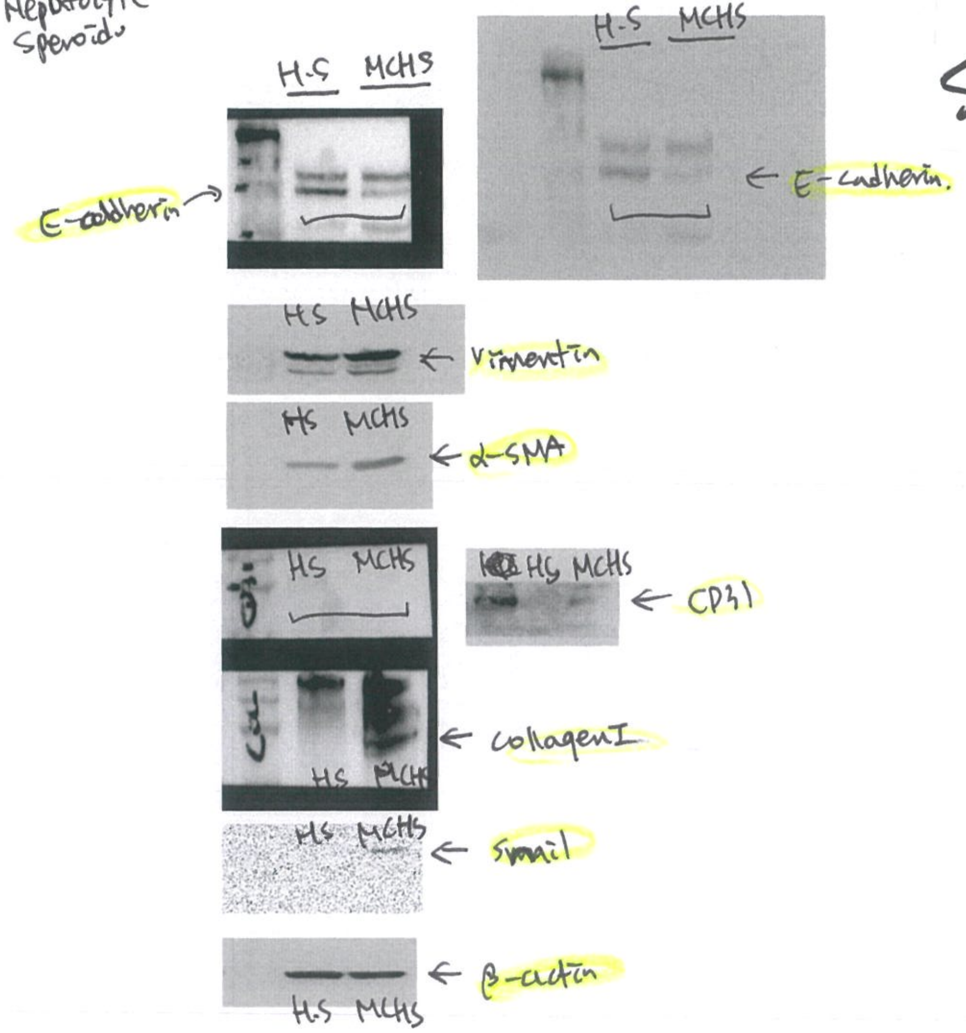

Supplementary figure 7

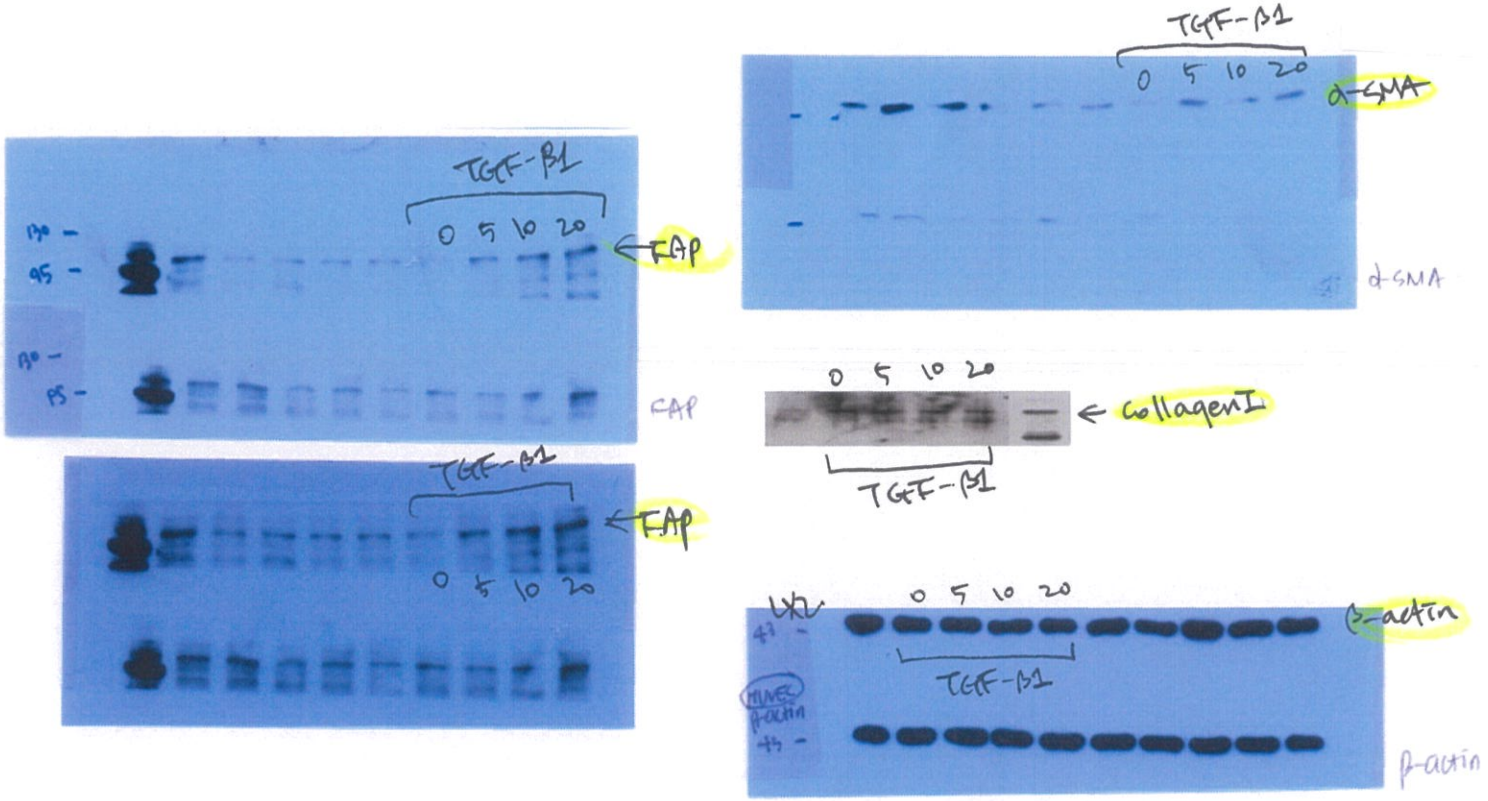

Supplement: Supplementary file 1 — Supplementary Figures. [file 41598_2021_90263_MOESM1_ESM.pdf]
